# Supplementary material for: Giant near-field nonlinear electrophotonic effects in an angstrom-scale plasmonic junction
Source: Nat Commun. 2026 Jan 24;17:2012. doi: 10.1038/s41467-026-68823-4 (PMC12936104; doi:10.1038/s41467-026-68823-4)
Supplement: Supplementary file 1 — Supplementary Information [file 41467_2026_68823_MOESM1_ESM.pdf]

Supplementary Information:

Giant near-field nonlinear electrophotonic effects in an angstrom-scale plasmonic junction

Shota Takahashi[1], Atsunori Sakurai[1,2]*, Tatsuto Mochizuki[1,2], and Toshiki Sugimoto[1,2]*

[1] Institute for Molecular Science, National Institutes of Natural Sciences; Okazaki, Aichi 444-8585, Japan.

[2] Graduate Institute for Advanced Studies, SOKENDAI; Okazaki, Aichi 444-8585, Japan.

*Corresponding authors. Email: asakurai@ims.ac.jp; toshiki-sugimoto@ims.ac.jp

Contents

Supplementary Note 1. The change in the surface morphology caused by SAM formation............3

Supplementary Note 2. Spectra of excitation light 4

Supplementary Note 3. Estimation of the absolute tip–substrate gap distance................................5

Supplementary Note 4. Fixing the tip–substrate distance for the measurements of voltage dependences 7

Supplementary Note 5. Forward-scattered TE-SHG signals 9

Supplementary Note 6. The stability of tip-enhanced nonlinear optical signals............................10

Supplementary Note 7. Conversion efficiencies of TE-SHG and TE-SFG responses....................12

Supplementary Note 8. STM images with and without laser irradiation—Negligibly small optical damage of the tip produced by excitation laser—..13

Supplementary Note 9. The absence of bias-induced structural change in MBT SAM 14

Supplementary Note 10. Origin of near-field nonlinear optical signals 16

Supplementary Note 11. Sample bias dependence of TE-SHG under constant tunneling current...18

Supplementary Note 12. Voltage dependent tip-enhanced nonlinear optical responses observed for different tips. 20

Supplementary Note 13. Fundamental mechanisms of TE-SHG and TE-SFG processes—Broadband optical response spanning infrared to visible region—...21

Supplementary Note 14. Spatial distributions of electric fields, radiation efficiencies, and charge densities within the gap 28

Supplementary Note 15. Influences of spatial distributions of electric fields on near-field nonlinear optical effects 33

Supplementary References...36

Supplementary Note 1. The change in the surface morphology caused by SAM formation.

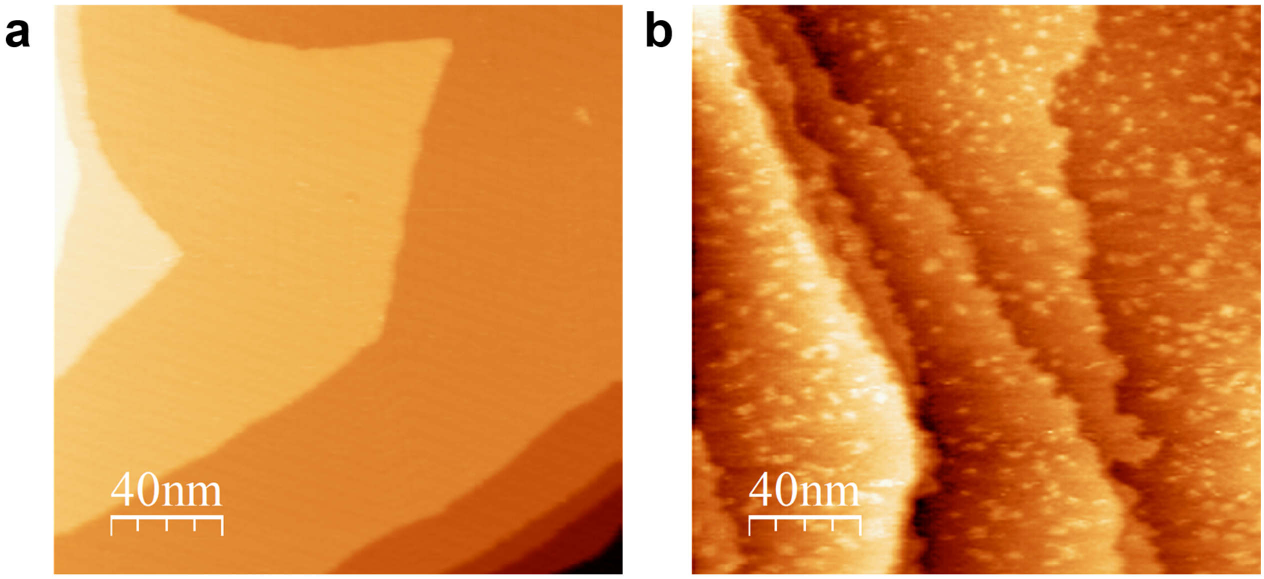

Supplementary Fig. 1 | The change in the surface morphology caused by SAM formation. STM images of Au(111) surface before (**a**) and after (**b**) immersion into ethanolic solution of MBT for 48 hours are shown. These images were obtained using a Au tip in constant current mode under sample bias voltage of −0.5 V. The tunneling current setpoints for (**a**) and (**b**) were 1 nA and 34.6 pA, respectively. The images were processed using WSxM software[1]. The substrate before immersion exhibited the straightened step edges and flat terraces. After immersion, the step edges became jagged, and patch-like protrusions appeared on the terrace. These characteristics are well-known and have also been reported in previous STM studies on aromatic thiolate SAMs[2–4]. Note that the protrusions in **b** were derived from Au adatom islands rather than from molecular aggregates[2–4]. The MBT monolayer is formed not only on the wide terrace region but also on the islands[2–4]. Therefore, the thickness of the SAM layer measured from the topmost gold surface is uniform throughout the surface and the tip-surface distance is always maintained constant during tip scanning at constant tunneling current and voltage.

Supplementary Note 2. Spectra of excitation light

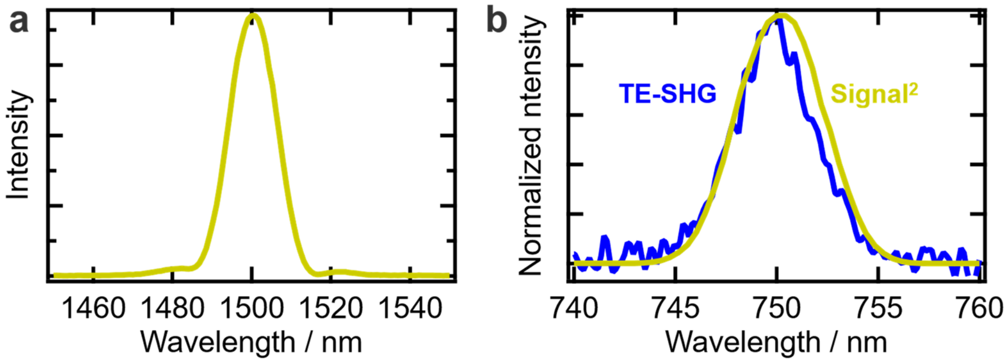

Supplementary Fig. 2 | Excitation source for TE-SHG experiments. a The spectrum of incident light ("signal" output from optical parametric oscillator (OPO) pumped by 1033-nm fundamental wave) used as excitation source of TE-SHG experiments. The central wavelength is located at 1500 nm. **b** Comparison of TE-SHG spectra (dark blue, same curve as shown in Fig. 2a in the main text) and the squared signal output spectrum (dark yellow). In the dark yellow curve, the wavelength axis values were halved to match the spectral positions. The spectral matching indicates that the shape of TE-SHG spectra is determined by the spectral distribution of the excitation light.

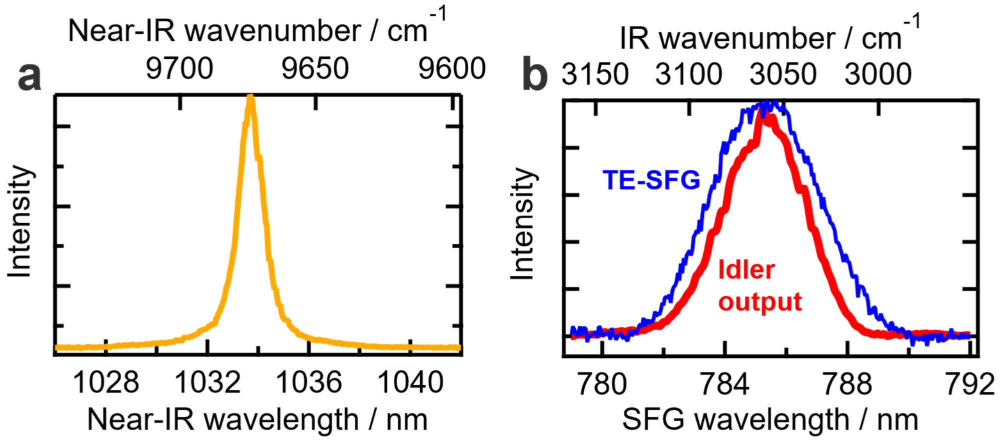

Supplementary Fig. 3 | Excitation sources for TE-SFG experiments. a The spectrum of near-IR incident light: "fundamental" output from Yb-fiber laser after passing through an air-spaced Fabry-Pérot etalon to narrow down the spectral width. **b** The spectrum of mid-IR incident light: "idler" output from 1030-nm pump OPO (red). Note that the mid-IR wavenumber is displayed in the top axis, and the corresponding values of the top axis are converted from the mid-IR wavenumber to the SFG wavelength and displayed in the bottom axis. For comparison, TE-SFG spectra (dark blue, same curve as shown in Fig. 4c in the main text) is superimposed. The slightly broadened feature of TE-SFG spectra results from the spectral convolution of the mid-IR pulse with a narrowband 1033-nm pulse shown in **a**. The spectral matching indicates that the shape of TE-SFG spectra is determined by the spectral distribution of these two excitation lights.

Supplementary Note 3. Estimation of the absolute tip–substrate gap distance.

The absolute gap distance between the tip apex and metal surface (d) is an important experimental parameter for tip-enhanced nonlinear optical measurements. This gap distance was estimated by the $I_t - d$ curve (Supplementary Fig. 4) for the Au tip and the Au substrate covered with SAM, which was measured by monitoring the tunneling current value (I_t) with moving the tip from the initial position ($\Delta d = 0$) toward the substrate under constant sample bias ($V = 0.1$ V). In the present study, the position of $\Delta d = 0$ is defined by the condition of tunneling current setpoint (I_t) of 10 pA and sample bias (V) of 0.1 V. Although the initial stage of the approach curve ($|\Delta d| < 1.5$ Å) can be well fit with an exponential function, the approach curve exhibits deviation from the exponential behavior after reaching to $\Delta d = -1.5$ Å that gives I_t~0.12 nA. Similar results were reported and reasonably explained considering that the vacuum layer and the SAM layer have independent decay constants for the electron tunneling[5–7]. Therefore, the contact point of the tip apex and the SAM should be the point at $\Delta d = -1.5$ Å. Considering the thickness of the MBT molecular layer (~6 Å), the absolute distance between the Au tip and the Au substrate surface at this contact point ($\Delta d = -1.5$ Å) can be determined as approximately 6 Å.

Based on this estimation, we can determine the absolute gap distance corresponding to the TE-SHG spectrum measured at $V = 0.1$ V and $I_t = 0.5$ nA (light blue curve in Fig. 2a in the main text). According to Supplementary Fig. 4, the tip position defined by $V = 0.1$ V and $I_t = 0.5$ nA corresponds to the point at $\Delta d = -2.7$ Å, which is 1.2 Å below the contact point. In this case, the absolute distance between the Au tip and Au substrate surface can be estimated as 6 Å − 1.2 Å = 4.8 Å~5 Å, with the tip apex slightly penetrating into the SAM. It should be noted that such direct contact conditions can sometimes give rise to additional signal enhancement effects arising from chemical bonding formation between the tip and sample[8]. However, since the terminal methyl group of MBT molecules is chemically inert, and its interactions with the tip are too weak to form chemical bonds, such additional enhancement should be negligibly small in our measurement conditions. Indeed, even when the metal–to–metal distance reached ~5 Å where the tip–molecule contact was expected, we did not observe any significant increase in signal intensity (the light blue curve in Fig. 2a in the main text). Therefore, the contribution of contact-induced chemical enhancement can be disregarded in this study.

Then, when the sample bias was increased from 0.1 V to 0.75 V under a constant tunneling current mode (0.5 nA), the tip–substrate gap distance was elongated by 2.1 Å (Fig. 2b in the main text). Therefore, the absolute tip–substrate gap distance at $V = 0.75$ V and $I_t = 0.5$ nA (dark blue curve in Fig. 2a in the main text) can be estimated as 4.8 Å + 2.1 Å = 6.9 Å~7 Å.

The absolute gap distances corresponding to the bias-dependent TE-SFG spectra measured under the constant tunneling current of 0.25 nA (Fig. 4c in the main text) can also be estimated in a similar way. According to Supplementary Fig. 4, the tip position defined by $V = 0.1$ V and $I_t = 0.25$ nA corresponds to the point at $\Delta d = -2.0$ Å, which is 0.5 Å below the contact point. In this case, the absolute distance between the Au tip and Au substrate surface can be estimated as 6 Å − 0.5 Å = 5.5 Å. Then, when the sample bias was increased from 0.1 V to 0.25 V under a constant tunneling current mode (0.25 nA), the tip–substrate gap distance was elongated by 1.2 Å

(Supplementary Fig. 5). Therefore, the absolute tip–substrate gap distance at $V = 0.25$ V and $I_t = 0.25$ nA (cyan curve in Fig. 4c in the main text) can be estimated as 5.5 Å + 1.2 Å = 6.7 Å. Similarly, according to the $d - V$ curve in Supplementary Fig. 5, the distances at V = 0.5 V (sky blue curve in Fig. 4c) and V = 0.75 V (dark blue curve in Fig. 4c) under $I_t = 0.25$ nA are estimated as 6.7 Å + 0.9 Å = 7.6 Å and 7.6 Å + 0.6 Å = 8.2 Å, respectively.

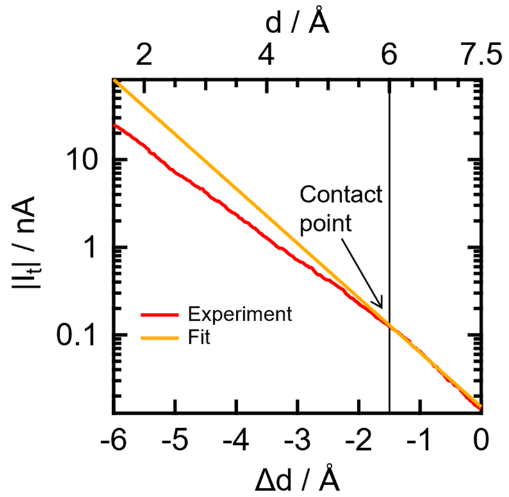

Supplementary Fig. 4 | $I_t - d$ curve for the tip–substrate gap. Variation of the tunneling current (I_t) with tip movement ($I_t - d$ curve) were obtained at the sample bias (V) 0.1 V. I_t is shown on a logarithmic scale. The initial position ($\Delta d = 0$) is defined by the conditions of $I_t = 10$ pA and $V = 0.1$ V. The absolute tip–substrate distance d is displayed in the top axis. The orange line is the fitting curve with an exponential function for the initial stage of the experimental $I_t - d$ curve.

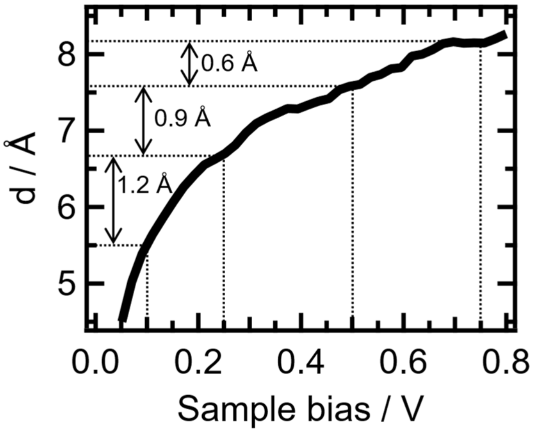

Supplementary Fig. 5 | Bias-induced change in the tip-sample distance. The absolute tip-sample distance (d) obtained at a constant tunneling current of 250 pA is plotted as a function of the sample bias. The tip–substrate distance at the feedback loop parameters of I_t = 250 pA and V = 0.1 V is 5.5 Å. The bias increase elongates the tip–substrate distance. Specifically, the distances at 0.25 V, 0.5 V, and 0.75 V are 6.7 Å, 7.6 Å, and 8.2 Å, respectively, which correspond to the experimental conditions where the TE-SFG spectra in Fig. 4c in the main text were obtained.

Supplementary Note 4. Fixing the tip–substrate distance for the measurements of voltage dependences

In the main text, we investigated the dependence of TE-SHG and TE-SFG intensities on applied voltages. In these experiments, it was critical to minimize the voltage-dependent changes in the gap distance between the tip apex and metal surface (d) to reduce the variation of field enhancement strength and isolate purely voltage-induced effects. The relationship between tip–substrate distance d, sample bias V, and tunneling current I_t can be described based on the Landauer theory of the conductance quantum as follows[9]:

$$\frac{I_t}{V} = G_0 \exp(-2\kappa d), \quad (1)$$

where G_0 and κ represent the quantum conductance and tunneling decay constant, respectively. Supplementary Equation (1) indicates that keeping tip–substrate distance (d) across various STM voltages (V) requires the tuning of the tunneling current setpoint (I_t) in synchronization with STM voltage sweeps. Therefore, prior to the voltage-dependent TE-SHG/TE-SFG measurements, we identified the combinations of V and $|I_t|$ values that can preserve gap distances using the following procedure.

Supplementary Fig. 6a shows typical STM $d - V$ curves obtained for MBT SAM on Au substrate, with two distinct $|I_t|$ values (60 pA and 600 pA). We here consider a horizontal line at d = 7 Å, the distance where the tip is placed 1 Å above the MBT molecules. The intersection points of this line with the $d - V$ curves correspond to the combination of V and $|I_t|$ setpoints that can maintain the tip–substrate distance at d~7 Å. For example, in Supplementary Fig. 6a, four such combinations are derived (orange cross marks): (−0.754 V, 600 pA), (−0.142 V, 60 pA), (+0.147 V, 60 pA), (+0.755 V, 600 pA). Using these combinations, the tip–surface distance d can be effectively kept constant although the voltage is varied.

By repeatedly measuring $d - V$ curves for various $|I_t|$ setpoints, we determined multiple combinations of V and $|I_t|$ values by identifying their intersections with the horizontal line at d = 7 Å (Supplementary Fig. 6b). Plotting these combinations produces the calibration curve ($I_t - V$) along which d is kept at 7 Å (Supplementary Fig. 6c). The voltage-dependent TE-SHG experiments shown in Fig. 3 in the main text were performed by selecting V and $|I_t|$ values along this calibration curve. The explicit V and $|I_t|$ values employed in our TE-SHG measurements are listed in Supplementary Table 1.

Additionally, as shown in Supplementary Figs. 6d and e, a similar calibration was performed again prior to the voltage-dependent TE-SFG experiments (Fig. 4d and e in the main text). The difference of $d - V$ curves shown in Supplementary Figs. 6b and e and the resultant calibration curve (Supplementary Figs. 6c and S5e) can be attributed to the variation in the tip apex structures: the difference in the tips influenced the $d - V$ characteristics of the gap and altered the appropriate combination of V and $|I_t|$ required to maintain a constant d. The explicit V and $|I_t|$ values adopted in our TE-SFG measurements are also listed in Supplementary Table 1.

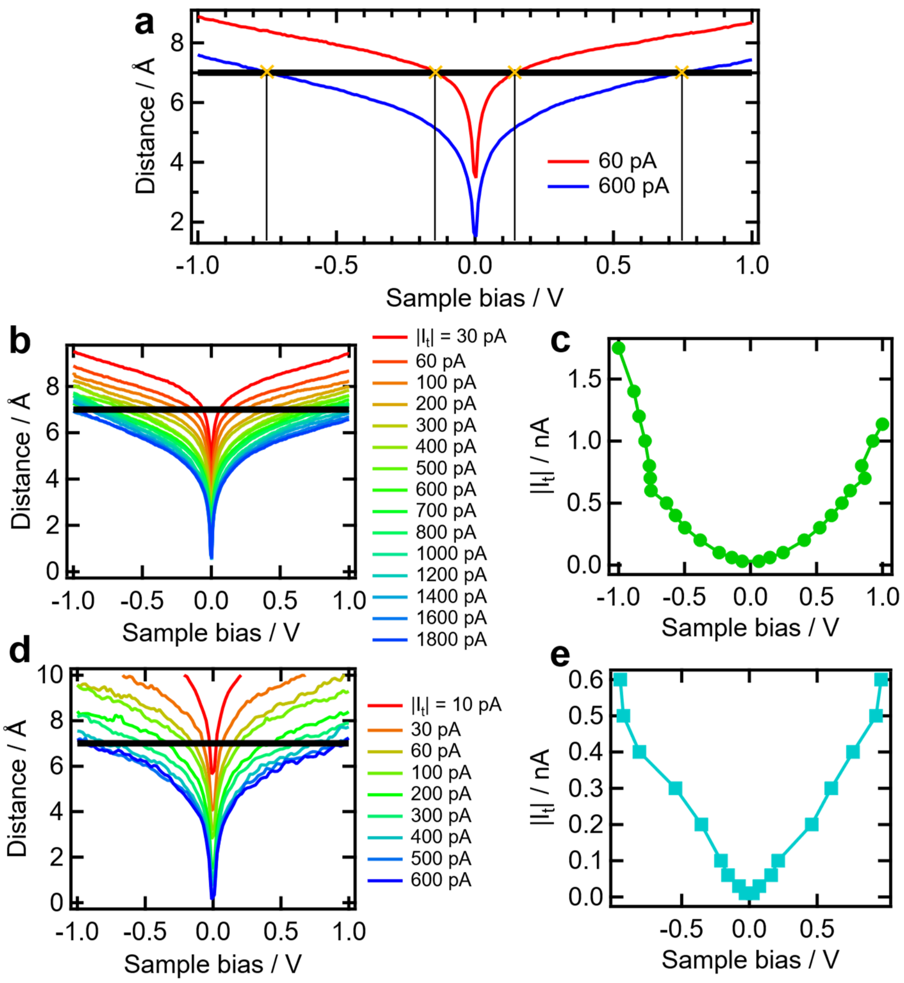

Supplementary Fig. 6 | Deducing V and $|I_t|$ values to fix the tip–surface distance. a Typical STM $d - V$ curves obtained for MBT SAM on Au substrate by using two different $|I_t|$ values (60 pA and 600 pA). The black horizontal line indicates the tip–surface distance of 7 Å. The orange cross marks represent the intersections between the line of $d = 7$ Å and $d - V$ curves. The combinations of V and $|I_t|$ setpoints at these intersections allow us to maintain d~7 Å. **b, d** $d - V$ curves for various $|I_t|$ setpoints. The black horizontal line indicates the tip–surface distance of 7 Å. The data in **b** and **d** were obtained with the tips used for voltage-dependent TE-SHG and TE-SFG experiments, respectively. **c, e** The $I_t - V$ calibration curve obtained by tracking the intersection points of $d = 7$ Å line with the $d - V$ curves. The voltage-dependent TE-SHG and TE-SFG measurements shown in the main text were performed by selecting the pairs of V and $|I_t|$ values along the curves in **c** and **e**, respectively.

Supplementary Table 1 Tunneling current setpoints at different sample biases to fix the tip–substrate distance at d ~ 7 Å

Bias	-1 V	-0.75 V	-0.5 V	-0.25 V	-0.1 V	0.1 V	0.25 V	0.5 V	0.75 V	1 V
TE-SHG	1750 pA	595 pA	304 pA	111 pA	47.2 pA	44.6 pA	102 pA	277 pA	591 pA	1135 pA
TE-SFG	-	381 pA	254 pA	127 pA	50.8 pA	50.8 pA	127 pA	254 pA	381 pA	-

Supplementary Note 5. Forward-scattered TE-SHG signals

Here, we briefly discuss the characteristic features of the forward-scattered TE-SHG signals by comparing them with the backward-scattered signals shown in the main text. Supplementary Fig. 7 shows the spectra obtained in the forward-scattering geometry under the irradiation of 10 pJ excitation light, which were measured simultaneously with the data shown in Fig 2a in the main text. When the tip–substrate distance was approximately 30 nm, the coherent far-field SHG signal was observed in the forward-scattering direction (gray curve in Supplementary Fig. 7), whereas no appreciable backward-scattered signal was observed (gray curve in Fig. 2a in the main text). These results allow us to exclude the possibility of the dominant effect of the tip plasmons excited at the Au tip apex alone, because if the tip plasmons could facilitate the appreciable level of enhanced SHG, the signals should be observed both in the forward- and backward-scattering geometry due to the dipole-like pattern of the plasmonic radiation. Therefore, the exclusive observation of forward-scattered SHG clearly indicates that the contribution from the tip plasmons excited at the Au tip apex alone is negligibly small and below the detection threshold. Such negligibly small contribution from the tip plasmon is more quantitatively supported by our theoretical calculation in Supplementary Fig. 18, which reveals that the field enhancement strength and radiation efficiency under the 30-nm tip–substrate distance are more than one order of magnitude weaker than those of angstrom-scale junction plasmon (see Supplementary Note 13 for details).

Then, the SHG intensity increased when the tip-substrate distance was reduced from 30 nm to ~5 Å (light blue curves in Supplementary Fig. 7 and Fig. 2a in the main text). As discussed in the main text, this is the manifestation of optical enhancement effect caused by the formation of plasmonic nanocavity between the substrate and the apex of the tip. Moreover, similarly to the backward-scattered signals shown in Fig. 2a in the main text, the forward-scattered TE-SHG also further increased when the sample bias (V) was increased from 0.1 V to 0.75 V (dark blue curve). Therefore, except for the existence of normal far-field signal, forward-scattered TE-SHG signals also exhibit similar enhancement behavior and voltage response.

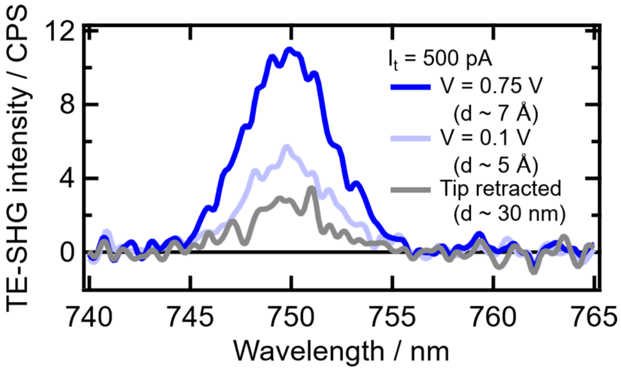

Supplementary Fig. 7 | The spectra of forward-scattered TE-SHG. These data were obtained at 1500-nm excitation and at sample biases of 0.1 V (light blue, d∼5 Å) and 0.75 V (dark blue, d∼7 Å) with a constant tunnelling current of 500 pA under room temperature and ultra-high vacuum (<1×10^{-7} Pa) condition. Gray curve indicates the signal obtained when the substrate was retracted enough from the tip (d∼30 nm) to deactivate the plasmonic enhancement effects.

Supplementary Note 6. The stability of tip-enhanced nonlinear optical signals

In this section, we discuss the stability of tip-enhanced nonlinear optical signals by examining the individual results of repeated measurements. The voltage dependence of TE-SHG intensities shown in Fig. 3 in the main text was obtained by multiple measurements repeated three times. The results of those three measurements are individually presented in Supplementary Fig. 8. Importantly, the different voltage sweeping orders were employed in each measurement. In the first measurement, the voltage was swept sequentially from −1 V to +1 V (filled orange squares). In the second measurement, the voltage was changed in the reverse direction, from +1 V to −1 V (open blue diamonds). In the third measurement, the voltage was initially swept from −0.1 V to −1 V, followed by a switch to +1 V and then sequentially reduced to +0.1 V (open green triangles). Despite these varied voltage sweeping sequences, the TE-SHG signals consistently exhibited a quadratic dependence on the applied voltage. This clearly indicates the minimal optical damaging effects on the tip during the measurements and ensures the reliability of our experimental results. Note that the error bars shown in Fig. 3 in the main text were obtained through these three measurements.

The stability of TE-SFG signals was also ensured in a similar way. The voltage dependence of TE-SFG intensities shown in Fig. 4d and e in the main text was obtained by sweeping the voltage in a random order. The explicit sequence of the voltage sweeping is displayed in Supplementary Table 2. Despite such random-order voltage sweeping, the TE-SFG signals exhibited clear quadratic dependence on the applied voltage. Note that under the individual bias voltages, 30-second signal accumulation was repeated ten times. The error bars shown in Fig. 4e in the main text were obtained through these ten measurements. More importantly, after performing measurements for eight different voltage values, we switched the voltage to the initial value (+0.75 V) again and confirmed that the TE-SFG intensity was reproduced (Supplementary Fig. 9). It should also be noted that this verification of the signal stability was performed under air atmosphere at room temperature. On the basis of the high reproducibility under the ambient conditions, we can conclude that the quadratic behavior is not due to accidental temporal change but is the manifestation of the intrinsic field-effect modulation of angstrom-scale gap structures that is operable even under the ambient conditions.

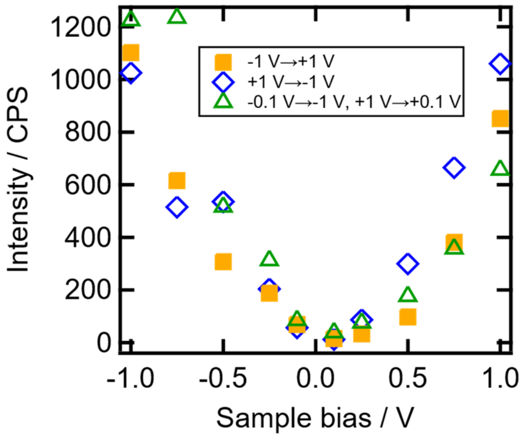

Supplementary Fig. 8 | Stability of TE-SHG measurements. The voltage-dependent changes in the TE-SHG intensity observed for three different voltage sweeping schemes are shown.

Supplementary Table 2 Voltage sweeping order employed in the voltage dependence measurements of TE-SFG.

Measurement order	1	2	3	4	5	6	7	8	9
Voltage / V	+0.75	−0.10	+0.25	+0.50	−0.25	−0.75	−0.50	+0.10	+0.75

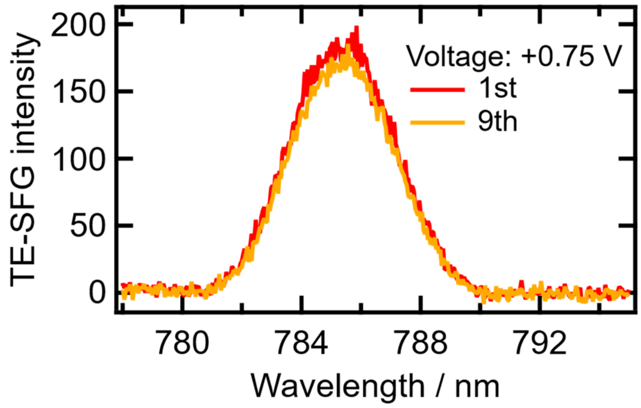

Supplementary Fig. 9 | Stability of TE-SFG measurements. TE-SFG spectra obtained under +0.75 V sample bias voltage. The measurements were performed in ambient condition. The red curve represents the initially obtained result (measurement order 1 in Supplementary Table 2). The orange curve represents the result finally obtained after performing measurements for eight different voltage values (measurement order 9 in Supplementary Table 2). Note that the TE-SFG spectrum at +0.75 V shown in Fig. 4d in the main text was given by the average of these two spectra.

Supplementary Note 7. Conversion efficiencies of TE-SHG and TE-SFG responses

In this section, we discuss the conversion efficiencies of TE-SHG and TE-SFG responses of the SAM-adsorbed gold substrate (Supplementary Fig. 10). Based on the FF-SHG intensity spectrum shown in Supplementary Fig. 7, the conversion efficiency of FF-SHG is estimated to be on the order of 10^{-14}. In contrast, the TE-SHG and TE-SFG exhibited higher efficiencies on the order of 10^{-11} and 10^{-12}, respectively (Supplementary Fig. 10), indicating that the nonlinear optical efficiency is enhanced by near-field electric field confinement. Importantly, this enhancement occurs despite the extremely small number of molecules involved in the TE-SHG/TE-SFG processes: based on the ~10-nm-scale field enhancement area (Supplementary Fig. 20) and the previously reported molecular density of the MBT SAM ($\sim 4 \times 10^{14}$ cm^{-2})[10], only ~10^3 molecules are estimated to contribute to TE-SHG/TE-SFG process. In contrast, the FF-SHG process should involve at least ~10^9 molecules within the micrometer-scale optical focus spot. Thus, while the absolute efficiencies are low, the per-molecule efficiency in the near-field scheme (10^{-14}–10^{-15}) is significantly higher than that in the far-field condition (~10^{-23}), clearly demonstrating the crucial role of the near-field enhancement (10^9–10^8) in the nonlinear optical generation processes. It should be noted that ~10^3 molecules are still too few to satisfy macroscopic phase-matching conditions, and the TE-SHG/TE-SFG signals under the present experimental conditions can therefore be approximated as originating from single-dipole radiation[11].

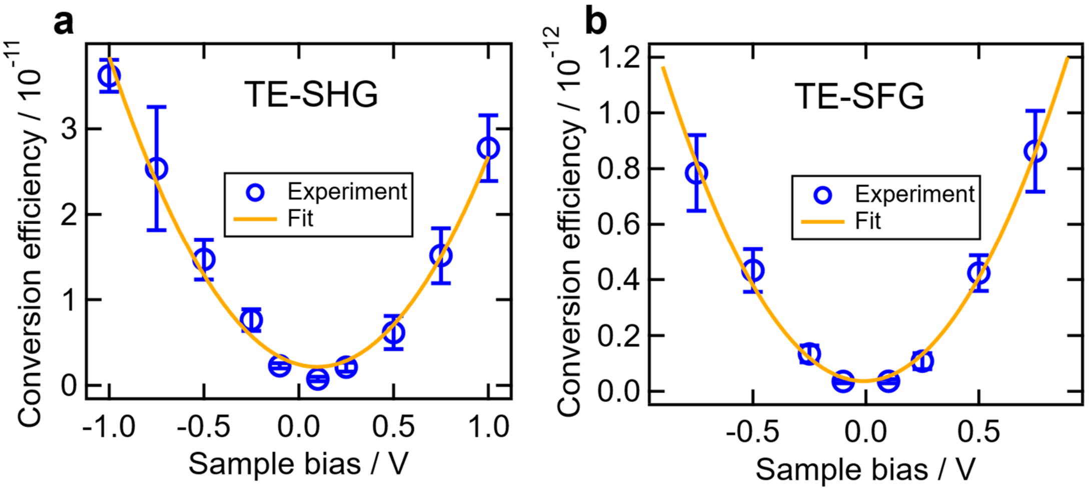

Supplementary Fig. 10 | Conversion efficiencies of TE-SHG (a) and TE-SFG (b) processes plotted against the sample bias. The conversion efficiency data presented in panel (**a**) were obtained by converting the photon counts used to evaluate the modulation depth in Fig. 3 in the main text into optical power and normalizing them by the incident power. The data in panel **b** were calculated in the same manner from the measurements shown in Fig. 4e in the main text. The error bars in panels **a** and **b** represent the standard error of the mean determined by three and ten independent repetitions of the same voltage-dependent measurement, respectively.

Supplementary Note 8. STM images with and without laser irradiation—Negligibly small optical damage of the tip produced by excitation laser—

To ensure that the exposure to excitation laser does not affect or damage the structure of the tip apex, we compared STM images of MBT-adsorbed Au(111) surface with and without 0.5 mW near-IR pulse irradiation (Supplementary Fig. 11). Although the STM images shown in Supplementary Figs. 11a and b were obtained at different locations on the substrate, the noise levels on both images were sufficiently low and angstrom-scale Au monoatomic step structures whose heights are consistent with previously reported values[12–14] were clearly captured (Supplementary Figs. 11c and d). Therefore, the fact that we were able to obtain such stable and high-quality STM images under laser illumination strongly supports the conclusion that the tip apex remains structurally stable during the measurements.

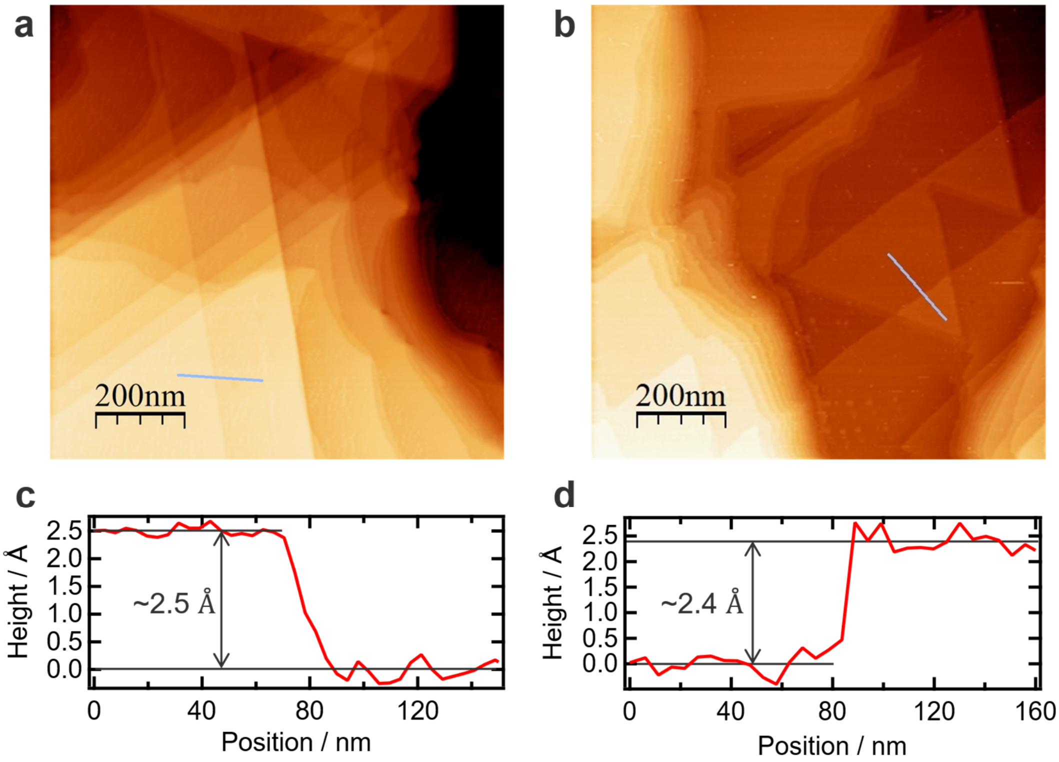

Supplementary Fig. 11 | The effect of laser irradiation on STM measurements. STM images of Au(111) with the adsorption of MBT SAM obtained without (**a**) and with (**b**) irradiating the tip-substrate gap region by near-IR excitation laser for TE-SHG experiments (50 MHz, 1500 nm, 0.5 mW). These STM images were measured at different locations on the substrate. The combinations of the bias voltages and tunneling current set points for (**a**) and (**b**) were (0.1 V, 1 nA) and (1 V, 0.2 nA), respectively. Both images clearly capture single-atom step structures with similar quality and low noise levels, confirming that the optical damage of the tip produced by excitation laser is negligibly small. **c, d**. Height profiles along the light blue lines in **a** and **b**, respectively. The STM images and height profiles in this figure were processed using WSxM software[1].

Supplementary Note 9. The absence of bias-induced structural change in MBT SAM

Previous experimental work demonstrated that MBT SAM on Au(111) substrate immersed in electrolyte solution exhibits drastic STM bias-induced structural transformation at approximately +0.3 V, accompanied by significant changes in the number and density of island-like structures within several minutes[10]. In contrast, in our ultra-high vacuum and air experimental conditions, such drastic structural change was not observed even when applying +1 V STM bias for more than 10 minutes (Supplementary Fig. 12). Therefore, we can reasonably assume that the molecular-level structure of the MBT SAM and its intrinsic second-order nonlinear optical susceptibility remain unchanged throughout our experiments.

The voltage-dependent TE-SHG/SFG measurements (Fig. 3 and 4 in the main text) were performed under these stable conditions, where no temporal structural changes in the SAM film were detected. Importantly, in these measurements, the bias values were varied in random orders (see Supplementary Note 6). Despite such non-sequential bias sweeping, the modulation curves exhibited a quadratic dependence on the applied voltage (Fig. 3 and 4 in the main text), and those experimental data were well-fit by using specific $\chi^{(2)}$ and $\chi^{(3)}$ values. This guarantees that no structural modifications occurred in the SAM during the measurements of voltage-dependent TE-SHG/SFG measurements, where the voltage values were swept within the range of –1 V to +1 V.

Notably, the absence of the structural modification of the tip and substrate indicates that dielectric breakdown within the gap and associated breakdown-induced damage to either the sample or the tip can be ruled out, even under the application of a strong electrostatic field (~10^9 V m^{-1}) across the gap. The impossibility of the dielectric breakdown can be correctly interpreted by considering the probability of the electron–gas collision within our angstrom-scale gap. Generally, the dielectric breakdown is induced by field emission of electrons from an electrode into gas phase, avalanche ionization of gas molecules, and ballistic acceleration of charge carriers within the interelectrode gap, leading to physical damage of the electrodes[15]. Thus, the collision process between electrons and gas molecules plays a key role in the emergence of dielectric breakdown. However, since the angstrom-scale tip–substrate distance employed in our experiments ensures the overlap of electronic wavefunctions at the tip and substrate surfaces, the interelectrode electron transfer occurs predominantly via the tunneling process, rather than ballistic field emission of electrons into the gas phase. This prevents electron–gas collisions that would otherwise trigger avalanche ionization of gas molecules, resulting in the absence of the dielectric breakdown. Moreover, considering the number density of gas molecules under ambient conditions (2.69×10^{19} cm^{-3}), the extremely small volume of the angstrom-scale gap (~10^{-20} cm^3) allows for the presence of at most a few gas molecules within the gap. This is insufficient to sustain the cascade ionization processes, making gas-phase electric breakdown physically impossible even under ambient experimental conditions. Therefore, the possibility of electric discharges within our angstrom-scale gap structure can be clearly ruled out, and any discharge-induced modification of the tip or substrate can be safely disregarded.

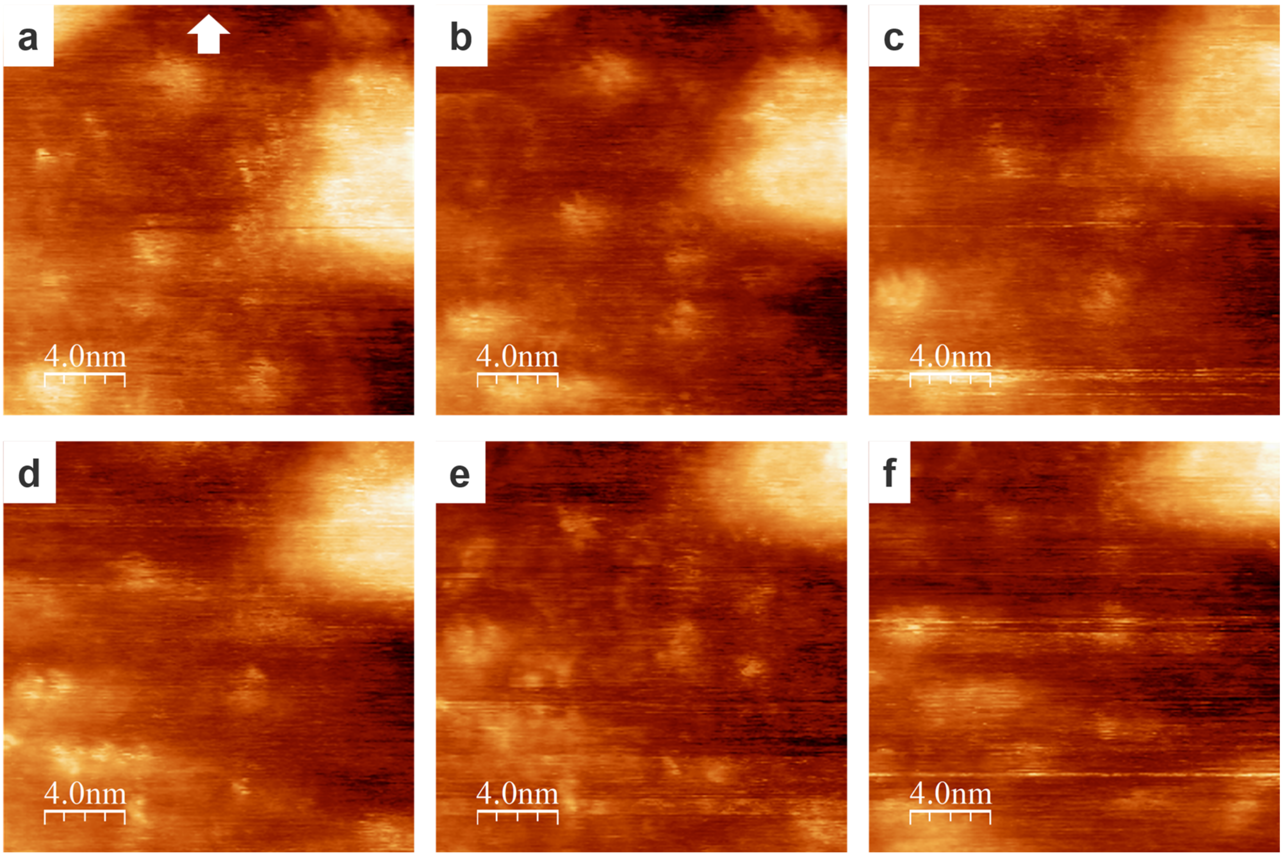

Supplementary Fig. 12 | Time-lapse STM images of MBT SAM on Au(111). The images were obtained sequentially from **a** to **f** under the sample bias of +1 V and tunneling current setpoint of 10 pA. The island-like structures that can be seen in each image correspond to the magnified view of the patch-like protrusions shown in Supplementary Fig. 1b. These structures were derived from Au adatom islands rather than from bumps of molecular aggregates, and the MBT monolayer is also formed on these islands[2–4]. The tip-substrate gap was not irradiated by excitation lasers. Since the acquisition time for each image was 105 seconds, it took more than 10 minutes to obtain all images. The thermal drift of piezoelectric stage where the sample was mounted gradually shifts the imaging region. The white arrow in **a** indicates the direction of the thermal drift of the piezoelectric stage. Note that these STM images were processed using WSxM software[1].

Supplementary Note 10. Origin of near-field nonlinear optical signals

To identify the origin of the near-field nonlinear optical signals presented in the main text, we conducted additional TE-SHG experiments using a bare Au(111) surface without adsorption of SAM molecules. As shown in Supplementary Fig. 13, the bare Au(111) surface also generated TE-SHG signals and exhibited large modulation depth on the order of 1000% V^{-1}, which is comparable to the case of the SAM-embedded gap. This finding indicates that the plasmonic gap structure itself plays a crucial role in the generation of tip-enhanced nonlinear optical signals and their field-induced modulation. We currently consider that the TE-SHG signals observed in the absence of the SAM film arise from the surface electrons of the Au tip and substrate, seeping out ~2 Å from their surfaces.

It is also noteworthy that the absolute TE-SHG signal intensity for the Au(111) substrate coated by SAM molecules was much higher than that of the bare Au(111) substrate (Supplementary Fig. 13a). Based on this result, we consider that the SAM film mainly contributes to the SHG signal generation. The difference in the absolute TE-SHG intensity for the Au substrates with and without the SAM can be understood from the spatial distribution of the electric field within the gap. In a previous study, electric field distribution within the gap was precisely calculated by incorporating atomic arrangements and charge distributions in an angstrom-scale gaps, predicting that the field enhancement at the interface between metal surface and vacuum region is generally weaker than that at the center of the gap region[16]. Thus, the SAM film within the gap should experience a substantially stronger electric field compared to the surface electrons. This pronounced difference in the strength of the electric field experienced by the media is likely a primary factor contributing to the variation in TE-SHG intensity shown in Supplementary Fig. 13a.

Additionally, the observed differences in TE-SHG intensity (Supplementary Fig. 13a) may also be caused by the differences in the effective nonlinear susceptibilities of the SAM film and the surface electron system. Moreover, in the case of bare Au (111) substrate, the electron wavefunctions spilling out from the tip apex and substrate surface are expected to more largely overlap compared with the SAM-covered configuration. Such overlap would enhance quantum plasmonic quenching effects[16–22], leading to a weaker near-field enhancement and consequently a smaller TE-SHG signal for the bare Au(111) substrate compared with the SAM-covered surface. A more quantitative analysis of these effects is beyond the scope of the present study and will be addressed in future work. Nevertheless, considering these possible factors, using the SAM film as the optical medium and positioning it within the gap region appears to be highly advantageous for enhancing TE-SHG signal.

Based on the above discussion, the overall mechanism governing the observed TE-SHG/TE-SFG and their bias-induced modulation can be comprehensively described as follows. While the nonlinear susceptibilities $\chi^{(2)}$ and $\chi^{(3)}$ originate from the medium within the gap (either the SAM or surface electron systems), plasmonic field enhancement remains indispensable for amplifying and detecting the near-field optical responses from the angstrom-scale gap region. Crucially, the exceptionally high bias-induced modulation depth (~2000% V^{-1}) observed in our study is fundamentally driven by the interaction between the third-order $\chi^{(3)}$ of the medium and the intense electrostatic field (E_{DC}) induced through voltage application. Although the absolute intensity of near-field nonlinear optical signals is determined by a complex interplay of various factors, including the spatial distribution of the electric field[16,23], the magnitude of nonlinear susceptibilities, and the

influence of quantum effects[22], the angstrom-scale plasmonic gap structure plays decisive roles in enabling and enhancing these near-field nonlinear optical processes.

Finally, it should be noted that, as theoretically predicted by Luca and Ciracì[24], the field-effect modulation of surface charge density may also contribute to the field-induced modulation of our TE-SHG/TE-SFG for the bare the Au (111) substrate. They proposed that by applying an external electrostatic field to a slab of heavily doped semiconductors, the density of free electrons in a very small region at the top surface is modulated and the drastic enhancement of free electron nonlinear optical responses becomes possible. They further predicted that such an effect would become more pronounced in a nanopatterned structure that supports plasmonic excitation and two orders of magnitude boost of free electron nonlinear optical response could be achieved. Their prediction is consistent with our experimental results for the angstrom-scale plasmonic gap, in which nonlinear optical signals were drastically enhanced in response to the application of static electric field (≤ 1 V). Therefore, we currently consider that their proposed mechanism would contribute to the observed bias-dependent giant TE-SHG modulation for the bare Au(111) surface (Supplementary Fig. 13). Although a comprehensive theoretical analysis is beyond the scope of this work, further investigation based on their theoretical framework and a quantitative comparison with our experimental results could substantiate this hypothesis, thereby unveiling a novel nonlinear electrophotonic modulation mechanism in quantum free-electron systems.

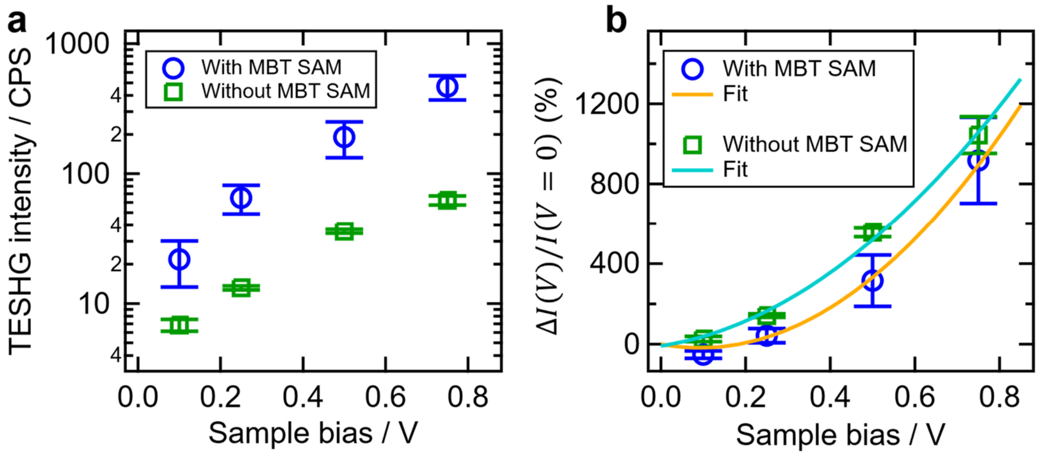

Supplementary Fig. 13 | TE-SHG intensity with and without SAM. a Sample bias dependence of absolute TE-SHG intensity obtained for Au(111) surface with (blue circles) and without (green squares) adsorption of MBT SAM. **b** The sample bias-dependent change in the TE-SHG intensity ($\Delta I(V)$) normalized by the signal intensity at $V = 0$ V ($I(V = 0)$). $I(V = 0)$ was obtained by averaging the intensities at $V = -0.1$ V and $V = 0.1$ V. Blue circles and green squares represent the data for Au(111) surface with and without adsorption of MBT SAM, respectively. The orange and light blue curves are the results of the curve fitting with quadratic functions conducted for the data indicated by blue circles and green curves, respectively. The voltage-dependent TE-SHG measurements of the Au(111) surface with and without a SAM were repeated three and ten times, respectively, and the resulting standard error of the mean is shown as error bars in panels **a** and **b**.

Supplementary Note 11. Sample bias dependence of TE-SHG under constant tunneling current. In the bias-dependent TE-SHG measurements presented in Fig. 3 in the main text, we varied not only the applied bias but also the tunneling current setpoint in the range of 40–1750 pA, allowing us to prevent the variation in the tip-substrate distance and maintain the field enhancement strength (see Table S1 for the explicit setpoint values). To check whether this current tuning influenced the observed clear quadratic bias dependence and ~2000% V^{-1} modulation of TE-SHG intensity, we performed control bias-dependent TE-SHG measurements under a constant tunneling current setpoint (Supplementary Fig. 14). As shown in Supplementary Fig. 14b, even in this constant-current condition, the TE-SHG intensity exhibited quadratic bias dependence, and the large modulation depth (~2000% V^{-1}) was still observed. These results indicate that the giant near-field nonlinear electrophotonic effects occur regardless of the tunneling current variations, demonstrating that the contributions of tunneling-current–driven processes, such as hot-carrier generation, inelastic scattering, impact ionization, photoluminescence, or electroluminescence, are negligibly small.

It should be noted that in this control experiment under the constant current mode, increasing the applied bias from 0.1 V to 1 V led to an elongation of the tip–substrate distance from ~4 Å to ~6 Å (Supplementary Fig. 14a). Based on the previous classical electrodynamic simulations, it is expected that the near-field enhancement strength significantly decreases during this tip–substrate distance elongation[17,21,25–28]. Nevertheless, the TE-SHG intensity markedly increased with the applied bias, and the pronounced quadratic bias dependence with a modulation depth of ~2000% V^{-1} was still observed under the constant current mode (Supplementary Fig. 14b). This result indicates that the expected distance-dependent variations in the field enhancement strength predicted by classical electromagnetic theory are essentially suppressed. This deviation from classical behavior can be attributed to quantum plasmonic quenching effects: at the gap distances of < 1 nm, the influences of quantum mechanical phenomena, such as electron spill-out from the metal surface and the overlap of electronic wavefunctions across the gap, begin to play roles and suppress the classically predicted field enhancement[16–22]. Particularly, in the gap distance range of 4–7 Å, these quantum suppression effects and the classically expected enhancement nearly cancel each other, making the overall field enhancement effectively independent of the tip–substrate distance[16–22]. Therefore, in the constant-current experiment shown in Supplementary Fig. 14, not only current-induced effects but also distance-dependent variations in field enhancement strength can be reasonably excluded. This allows us to clearly attribute the observed TE-SHG modulation to the voltage-induced nonlinearity represented by the $\chi^{(3)}E_{\mathrm{DC}}$ term.

Notably, further reduction of the tip–substrate distance below ~4 Å leads to a regime where quantum plasmonic quenching becomes dominant, resulting in a steep decrease in the electric field enhancement factors[16–22]. In contrast, the 4–7 Å regime represents a critical crossover region just before this steep decline, where the electric field enhancement is maximized[16–22]. Due to this compensation, the overall field enhancement factor remains nearly constant at its maximum value throughout this gap-size range, rendering it effectively independent of tip–substrate distance from 4 Å to 7 Å. Thus, the 4–7 Å distance range not only offers optimal field enhancement conditions for both TE-SHG and TE-SFG processes, but also provides an ideal regime for exclusively probing the intrinsic bias dependence of the giant electrophotonic response, distinct from variations in the near-

field enhancement.

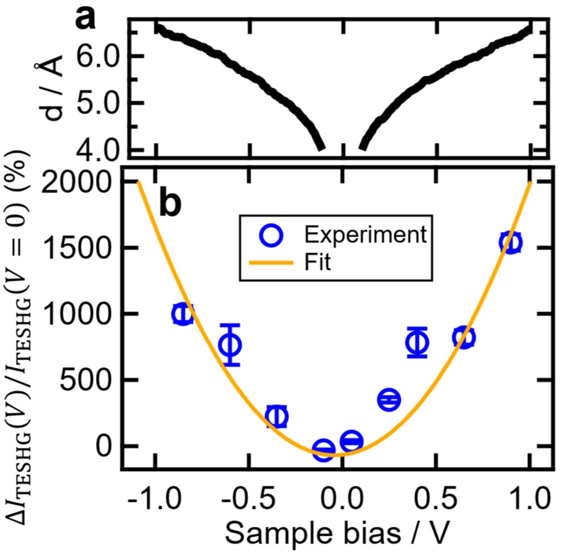

Supplementary Fig. 14 | Sample bias dependence of TE-SHG under constant tunneling current. **a** Sample bias dependence of the tip–substrate distance (d) with respect to the distance at feedback loop parameters of $V = 0.1$ V and $I_t = 500$ pA. The measurement was performed at a constant tunneling current of 500 pA. **b** The voltage dependence of TE-SHG intensity under constant tunneling current (500 pA). The error bars represent the standard error of the mean determined from ten independent repetitions of the same voltage-dependent measurement.

Supplementary Note 12. Voltage dependent tip-enhanced nonlinear optical responses observed for different tips.

We checked the reproducibility of the voltage dependence of TE-SHG and TE-SFG processes by measuring the intensities of TE-SHG and TE-SFG using three different tips shown in Supplementary Figs. 15a–c. Although the slight differences in the apex structures could give rise to differences in the electrostatic field distribution and hence the fewfold differences in the degree of bias dependent signal enhancement, we successfully confirmed that these tips exhibited strong plasmonic enhancement of nonlinear optical responses and similar voltage-dependent enhancement behaviors emerged (Supplementary Figs. 15d–f). This ensures not only the reproducibility of our results but also the generality of our concepts of giant electrical modulation of nonlinear optical processes based on an angstrom-scale plasmonic gap structure.

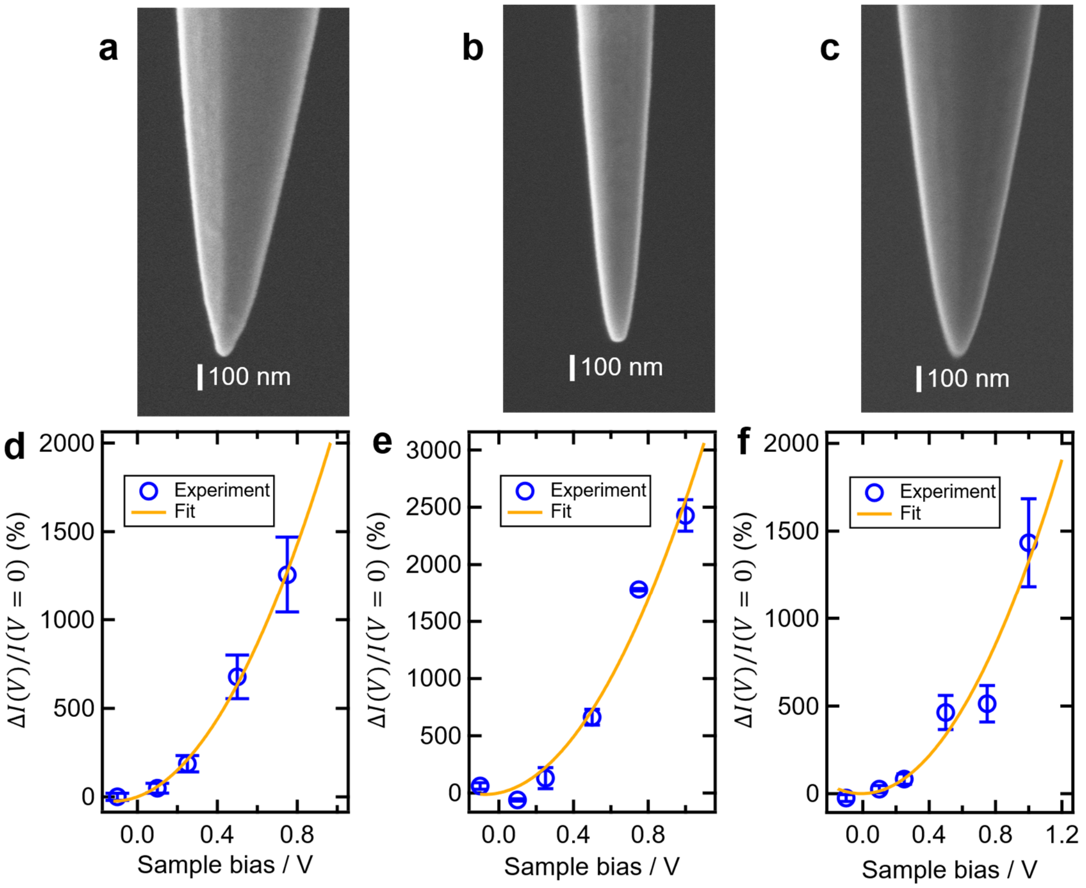

Supplementary Fig. 15 | Reproducibility of giant electrical enhancement of nonlinear optical processes with different tips. a–c Scanning electron micrographs of Au tips. These tips were independently fabricated and used in the experiments to ensure the reproducibility of giant electrical enhancement of nonlinear optical processes. **d–f** The voltage-dependent changes in the TE-SHG (**d**) and TE-SFG (**e, f**) output ($\Delta I(V)$) normalized by the signal intensities at $V = 0$ V ($I(V = 0)$). The orange curves are the result of curve fitting with a quadratic function. The data in panels **d**, **e**, and **f** were obtained by using the tips shown in **a**, **b**, and **c**, respectively. The data shown in panels **d** and **e** were obtained from ten independent repeated measurements, while those shown in panel f were obtained from three independent repeated measurements. The error bars represent the standard error of the mean calculated from these repeated measurements.

Supplementary Note 13. Fundamental mechanisms of TE-SHG and TE-SFG processes—Broadband optical response spanning infrared to visible region—

As discussed in the main text, we observed giant electrophotonic effects characterized by the quadratic voltage dependence and ~2000% V^{-1} modulation depth in TE-SHG and TE-SFG intensities. Since these nonlinear optical processes involve infrared-to-visible drastic frequency conversion between the incoming and outgoing light, a spectrally broad plasmonic enhancement that can simultaneously affect such separated frequencies is critical for realizing efficient generation of TE-SHG and TE-SFG. In the main text, however, we omitted the detailed description of the spectral properties of field enhancement strength in order to simplify the mathematical expression and exclusively focus on the effect induced by the electrostatic fields. Thus, in this section, we provide an overview of the electric-field enhancement mechanisms governing TE-SHG and TE-SFG processes by explicitly incorporating the plasmonic filed enhancement into the discussion[23,29].

SHG involves two-photon excitation (generation of nonlinear polarization $P^{(2)}$) and one-photon emission (radiation from $P^{(2)}$) processes. The excitation and radiation processes are simultaneously amplified at the angstrom-scale gap in STM through plasmonic field enhancement, leading to a substantial increase in SHG signals. Moreover, the third-order field-induced effect driven by the electrostatic field within the angstrom-scale gap (E_{DC}) contributes to the modulation of the SHG signals. Consequently, the overall TE-SHG intensity (I_{TESHG}) can be described by using the incident field enhancement factor ($K_{\mathrm{gap}} \equiv E_{\mathrm{gap}}/E_0$), the enhanced emission efficiency from $P^{(2)}$ (L_{gap}), and second- and third-order nonlinear optical susceptibility ($\chi^{(2)}$ and $\chi^{(3)}$) as follows[23]:

$$I_{\mathrm{TESHG}}(2\omega) \propto \left|\chi^{(2)} + \chi^{(3)} E_{\mathrm{DC}}\right|^2 \left|K_{\mathrm{gap}}(\omega) E_0\right|^4 \left|L_{\mathrm{gap}}(2\omega)\right|^2, \quad (2)$$

where ω represents the excitation frequency for TE-SHG. Note that since the amplitudes of K_{gap}, L_{gap}, and E_{DC} inherently depend on spatial positions within the gap (see Supplementary Note 14 for the spatial distributions of these factors), the overall TE-SHG intensity should be evaluated by weighting contributions from individual nonlinear polarizations generated over the whole gap region. However, to provide a simple physical understanding of the fundamental mechanisms of the TE-SHG process, we here exclusively focus on K_{gap} and L_{gap} values sampled at a single point directly beneath the tip apex, without considering their spatial distributions over the gap region. More rigorous theoretical treatments of TE-SHG intensities that explicitly incorporate the spatial distributions of K_{gap}, L_{gap}, and E_{DC} within the gap are provided in Supplementary Note 15.

Supplementary Equation (2) provides an expression for TE-SHG process that incorporates not only electrophotonic effect ($\chi^{(3)} E_{\mathrm{DC}}$) but also the spectral properties of the incident field enhancement ($K_{\mathrm{gap}}(\omega)$) and the signal emission efficiency ($L_{\mathrm{gap}}(2\omega)$). By denoting $\left|K_{\mathrm{gap}}(\omega) E_0\right|^2$ as I_{gap} and omitting $L_{\mathrm{gap}}(2\omega)$ term for simplicity, we arrive at Eq. 1 in the main text, representing a simplified expression where the electrophotonic effect on TE-SHG is more specifically featured. Here, to understand the fundamental mechanism governing TE-SHG process, we focus on the frequency-dependent terms in Supplementary Equation (2) rather than the electrophotonic term. Since both incident excitation light and emitted SHG light in our experiments are non-resonant with electronic/vibrational transitions of MBT molecules and gold, we can assume that the frequency

dependences of $\chi^{(2)}$ and $\chi^{(3)}$ are small. Therefore, the overall frequency profile of TE-SHG is approximated as

$$I_{\mathrm{TESHG}}(2\omega) \propto \left|K_{\mathrm{gap}}(\omega)\right|^4 \left|L_{\mathrm{gap}}(2\omega)\right|^2. \quad (3)$$

Supplementary Equation (3) indicates that the spectral properties of TE-SHG can be generally described by considering the field enhancement factor ($K_{\mathrm{gap}}(\omega)$) and the enhanced emission efficiency ($L_{\mathrm{gap}}(2\omega)$). Therefore, the underlying enhancement mechanism of the TE-SHG process can be understood by examining $K_{\mathrm{gap}}(\omega)$ and $L_{\mathrm{gap}}(2\omega)$.

The procedure for electromagnetic field simulations was described elsewhere[23,29]. Briefly, the finite-difference time-domain (FDTD) method[30–32] was adopted with commercial software (Lumerical FDTD, Ansys). The system investigated in the simulation consists of a gold tip positioned above a gold substrate in vacuum, representing the nanogap in our STM. The refractive index of gold was taken from the experimental values of Olmon *et al.*[33] Perfectly matched layer boundary conditions were used in all simulations to absorb all outgoing waves and eliminate light reflection. To evaluate the spectral properties of incident field enhancement (K_{gap}), we placed a monitor at the midpoint between the tip apex and the substrate surface to measure the electromagnetic field strength. A p-polarized Gaussian beam source with a waist of 2 μm was used to illuminate the nanogap at an incident of 55°. To reduce the computational cost, the calculation region was restricted to a two-dimensional plane parallel to the plane of incidence and containing the longitudinal axis of the tip.

To evaluate the radiation efficiency (L_{gap}), an oscillating dipole source perpendicular to the gold substrate, representing the nonlinear polarizations generated within the gap, was placed at the same position as the monitor for K_{gap}. The selection of the dipole source is verified based on our recent demonstration[11]: we experimentally and theoretically revealed that when the tip apex size is on the order of ~50 nm as in the present study (Fig. 1b in the main text), the generated nonlinear polarizations are highly dipole in nature, with no significant contributions from quadrupoles or higher-order multipoles[11]. Radiated electromagnetic field from the dipole was monitored at a position where the lateral and vertical distances from the dipole were 3000 and 2100 nm, respectively, corresponding to the reflection angle of 55° employed in our experiments. To reduce the computational cost, the calculation region was restricted to a two-dimensional plane perpendicular to the substrate surface and containing the point dipole source and the field monitor. Note that nonlocal or quantum-corrected models[16–22] were not explicitly considered in our simulations. Moreover, since the near-field enhancement factor within the tip–substrate gap was revealed to be essentially unaffected by the STM bias of ~1 V applied across a tip–substrate junction maintained at constant distance[34], the influence of the applied bias was not incorporated in our FDTD simulation.

In the following, we review previously reported spectral characteristics of the field enhancement factor and radiation efficiency[23,29] to facilitate a clear understanding of the broadband IR-to-visible near-field enhancement that is essential for the TE-SHG and TE-SFG processes. Initially, we started by modeling an STM tip as a nanosphere without a shaft[23,28,35–39] (Supplementary Fig. 16a), and the wavelength dependences of K_{gap} and L_{gap} calculated for this nanosphere-substrate system is shown in Supplementary Figs. 16b and c, respectively. The spectra have a single enhancement band in the visible region. This result aligns with a typical signature of the gap-mode plasmon excitation

localized between the nanogap.[36,40–42] However, in the infrared region, both of the K_{gap} and L_{gap} values seem to be too small to explain the enhancement of infrared-light-excited TE-SHG and TE-SFG. This implies that micrometer-scale tip shafts, which were not considered in the nanosphere-substrate system, play an important role in the enhancement of the infrared region.

To clarify the influence of the micrometer-scale tip shafts, we then performed more realistic FDTD simulations, in which an STM gap structure was modeled by a rounded cone-shaped gold tip with a longitudinal shaft (tip length: l) placed above a flat gold substrate (Supplementary Fig. 16d)[23]. The opening angle and curvature radius of the tip apex were 30° and 50 nm, respectively. Supplementary Fig. 16e shows the K_{gap} spectra calculated for various tip lengths. For short tips ($l \leq 150$ nm), the enhancement remained localized in the visible region, driven by the gap-mode plasmon resonance described above. In contrast, as the tip length is extended (particularly $l \geq 600$ nm), the broadband enhancement profile covering the broad near- and mid-IR region emerged. We confirmed that the calculation converged at $l = 15000$ nm and the broadband electric field enhancement remains at this shaft length. The results obtained for the long tips correspond to the actual experimental situation, where the Au tips with micrometer-scale lengths (Fig. 1b in the main text and Supplementary Figs. 15a–c) were used. Therefore, we can conclude that the field enhancement at the nanogap is highly effective over broad wavelength range encompassing visible, near-IR and mid-IR region. This drastic enhancement in the infrared region is a clear manifestation of the influence of the long tip shafts, whose origin can be attributed to the antenna effect caused by the collective oscillation of electrons over the entire tip[43–46].

On the other hand, in the radiation process, the effect of the tip shafts is not pronounced. As shown in Supplementary Fig. 16f, the spectra of L_{gap} were still limited to a single band in the visible domain regardless of the tip length l. Although the variations in the tip length cause slight differences in the strength and shape of the L_{gap} spectra, the wavelength range of efficient radiation from nanocavities is predominantly determined by gap-mode plasmons. This is because the time-averaged power of the vacuum propagating electromagnetic field radiated by the oscillating polarization is proportional to λ^{-4} and steeply decreases with wavelength, making the contributions from the antenna effects in the IR region less dominant.

Note that the influence of dipolar and quadrupolar plasmonic modes on the signal radiation process has been comprehensively discussed in a previous work[28]. Although a sharpened tip with an apex radius of < 5 nm supports a quadrupolar plasmonic mode, a larger tip with a radius of > 10 nm exhibits only dipolar plasmonic mode[28], indicating that the excitation of the quadrupolar plasmons is negligible for the larger tip apex on the order of several tens of nanometers. Since ~30-nm tip apex employed in our experiments (Fig. 1b in the main text) falls into this dipole-dominated regime, the near-field radiation process in this work should be mediated predominantly by dipolar gap-mode plasmons.

The spectral characteristics of K_{gap} and L_{gap} in the long tips (the blue curves in the bottom of Supplementary Figs. 16e and f) are key to understand the mechanism of infrared-excited TE-SHG process. Supplementary Fig. 17a shows the wavelength dependence of the TE-SHG intensity calculated using Supplementary Equation (3) and K_{gap} and L_{gap} spectra obtained for $l =$

15000 nm. This spectral profile indicates that the TE-SHG process is highly efficient over broad near-IR range encompassing $\lambda \geq 1100$ nm region. This spectrally broad effectiveness of TE-SHG is the consequence of the fact that the broadband enhancement of incident light in the near-to-mid-infrared region (K_{gap}) and efficient radiation in the visible-to-near-infrared region (L_{gap}) effectively cover the excitation and SHG radiation wavelength ranges, respectively. Thus, the SHG enhancement arises from the simultaneous amplification at ω (excitation) and 2ω (radiation) caused by the concerted operation of two distinct enhancement mechanisms: the antenna effects caused by micrometer-scale tip shafts enhance infrared excitation, while localized gap-mode plasmons intensify the radiation of second harmonics.

It should also be remarked that a similar discussion can be applied to the TE-SFG process. In analogy with Supplementary Equation (3), the frequency profile of TE-SFG can be described by the product of the enhancement factor and radiation efficiency:

$$I_{\mathrm{TESFG}}(\omega_{\mathrm{SFG}}) \propto \left|K_{\mathrm{gap}}(\omega_1)\right|^2 \left|K_{\mathrm{gap}}(\omega_2)\right|^2 \left|L_{\mathrm{gap}}(\omega_{\mathrm{SFG}})\right|^2, \quad (4)$$

where I_{TESFG} is the output TE-SFG intensity; ω_1 and ω_2 represent the two different frequencies of excitation light for TE-SFG; and ω_{SFG} represents the sum of ω_1 and ω_2. Similarly to the case of TE-SHG, Supplementary Equation (4) allows us to predict the wavelength dependence of TE-SFG intensity (Supplementary Fig. 17b), exhibiting the broadband effectiveness spanning not only near-IR but also mid-IR region. Therefore, the concerted effect of the antenna effects in the infrared region and the gap-mode plasmon in the visible region also governs the enhancement mechanism of TE-SFG process.

To further improve the TE-SHG and TE-SFG efficiencies, it is essential to tailor the enhancement spectrum such that the value of $\left|K_{\mathrm{gap}}(\omega_1)K_{\mathrm{gap}}(\omega_2)L_{\mathrm{gap}}(\omega_1+\omega_2)\right|^2$ terms in Supplementary Equations (3) and (4) are maximized. As discussed above and demonstrated in more detail in our previous work[23], the spectral profiles of K_{gap} and L_{gap} are highly sensitive to both nanometer- and micrometer-scale tip geometry. Specifically, the nanometer-scale curvature of the tip apex primarily determines the gap-mode plasmon resonance in the visible region, whereas the micrometer-scale surface geometry of the tip shaft governs the broader field enhancement at the IR region through the antenna effect[23]. Therefore, to improve the field enhancement strengths at both the visible and IR regions, simultaneous control of these different-scale structures through nanoscale adjustment of the apex curvature[47] and the introduction of grating patterns on the micrometer-scale tip shaft[48,49] is essential, leading to further improvement of the TE-SHG/TE-SFG efficiencies. Such fine control of tip geometries would be achieved by exploiting more sophisticated tip processing techniques, such as field-directed sputter sharpening for the tip apex[50] and focused ion beam processing for the tip shaft[51]. We believe that implementing these strategies to improve the overall near-field nonlinear optical efficiencies represents an important research direction for our future work, potentially leading to novel strategies to improve the conversion efficiency of near-field nonlinear optical processes.

Additionally, to examine the influences of tip plasmons generated in the tip-retracted conditions (gray curves in Fig. 2 in the main text and Supplementary Fig. 7), we expanded the tip–surface distance to 30 nm and calculated the spectra of the field enhancement factor $\left(\left|K_{\mathrm{gap}}\right|^2\right)$ and

the emission efficiency $\left(\left|L_{\mathrm{gap}}\right|^2\right)$ (Supplementary Fig. 18). For calculating the $\left|K_{\mathrm{gap}}\right|^2$ spectrum, the monitor for the electromagnetic field was placed 0.5 nm below the tip apex. As a result, the electric field enhancement (Supplementary Fig. 18a) and emission efficiency (Supplementary Fig. 18b) under the 30-nm-retracted condition were found to be more than one order of magnitude smaller than those in an angstrom-scale tip–substrate plasmonic gap. These values should be too weak to produce a detectable enhancement of nonlinear optical signals from nanoscale tip apex, thereby allowing us to safely disregard the contribution from tip plasmon-enhanced signals in our experiments.

Finally, to show the influence of the presence of the SAM layer on the field enhancement spectra, we performed additional electromagnetic field simulations incorporating 6-Å-thick SAM layer within the tip–substrate gap with 1-nm distance (Supplementary Fig. 19a). The refractive index of the SAM layer was set to be 1.2, which represents the typical value that has been used to calculate the optical responses of interfacial SAM layers[52–54]. As shown in Supplementary Figs. 18b and c, the presence of the SAM layer induces only minor changes in the spectral profiles of both field enhancement factor (Supplementary Fig. 19b) and radiation efficiency (Supplementary Fig. 19c). Therefore, although the above discussion on the field enhancement mechanisms is based on the vacuum gap, the same conclusion can be deduced even in the presence of the SAM layer within the gap.

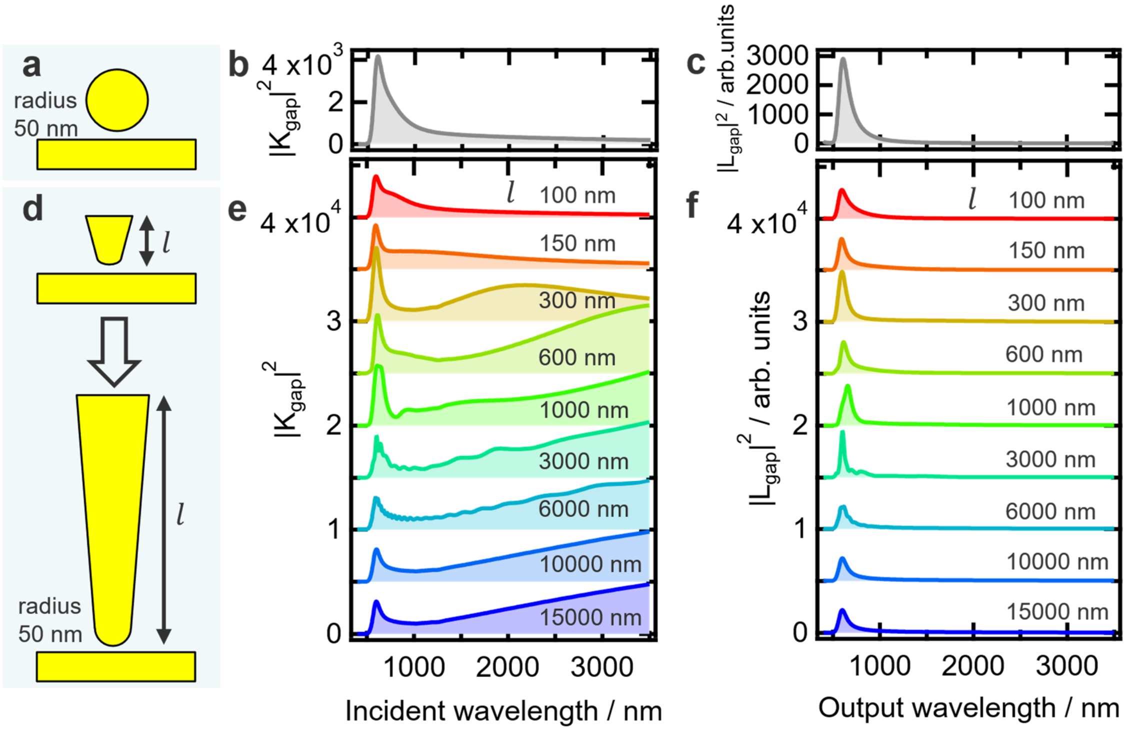

Supplementary Fig. 16 | Theoretical calculation of the field enhancement factor and the radiation efficiency revealing the mechanism of infrared-to-visible broadband nonlinear optical responses. a Schematic representation of nanosphere-substrate configuration. The radius of the nanosphere is 50 nm. **b** $\left|K_{\mathrm{gap}}\right|^2$ and **c** $\left|L_{\mathrm{gap}}\right|^2$ spectra of the nanogap in a nanosphere-substrate system calculated through the FDTD method. **d** Schematic representation of tip-substrate configuration. A rounded cone tip with a 30° opening angle and 50 nm radius of curverture was

adopted in the calculation. The tip length l was changed from 100 nm to 15000 nm. **e, f** Tip-length dependent (**e**) $|K_{gap}|^2$ and (**f**) $|L_{gap}|^2$ spectra of a tip–substrate nanocavity calculated through the FDTD method. The tip lengths are indicated in the figures and the tip–substrate distance d was taken as 1 nm for all these calculations. The calculation in **e** and **f** converged at $l = 15000$ nm. The results obtained for longer tips corresponds to the actual experimental conditions where the micrometer-scale Au tips (Supplementary Figs. 15a–c) were used.

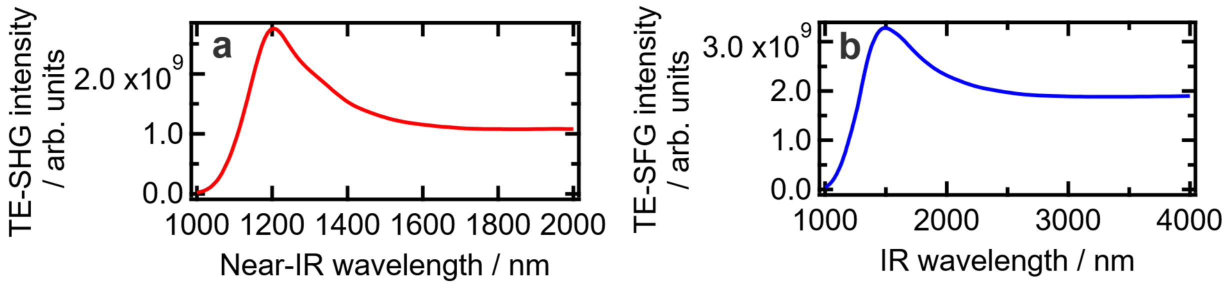

Supplementary Fig. 17 | Spectral properties of tip-enhanced second-order nonlinear optical processes. The excitation wavelength dependence of TE-SHG (**a**) and TE-SFG (**b**) calculated based on Supplementary Equations (3) and (4), respectively. In calculating these spectral properties, $|K_{gap}|^2$ and $|L_{gap}|^2$ spectra obtained for $l = 15000$ nm tip (Supplementary Figs. 16e and f) were adopted. Note that the horizontal axis in b represents one of the two excitaiton wavelengths of SFG. Another excitation wavelength was fixed at 1033 nm, which corresponds to the central wavelength of near-IR excitation pulses used in our experiments.

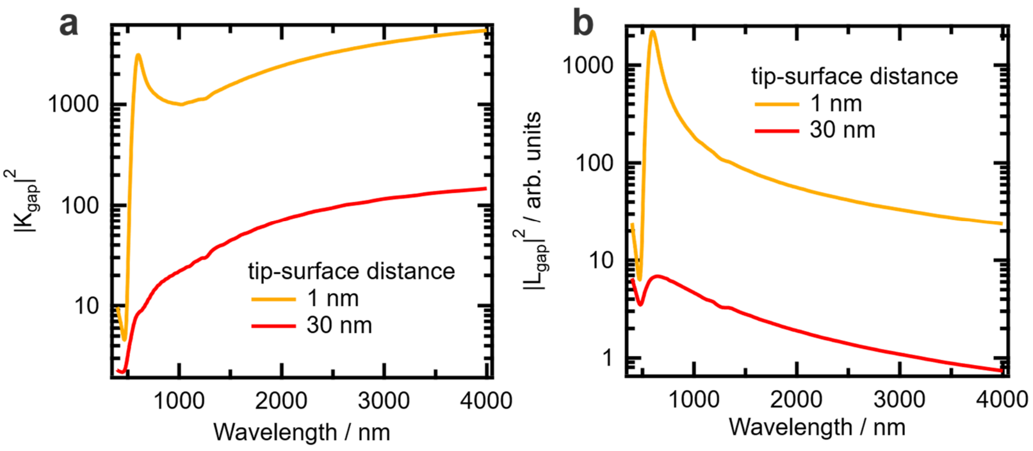

Supplementary Fig. 18 | Calculation of field enhancement caused by a tip plasmon. The spectra of field enhancement factor $|K_{gap}|^2$ (**a**) and emission efficiency $|L_{gap}|^2$ (**b**) calculated for 1-nm (orange) and 30-nm (red) tip-substrate gap distances. The orange curves in **a** and **b** are identical to those shown in Supplementary Figs. 16e and f obtained when $l = 15000$ nm, respectively.

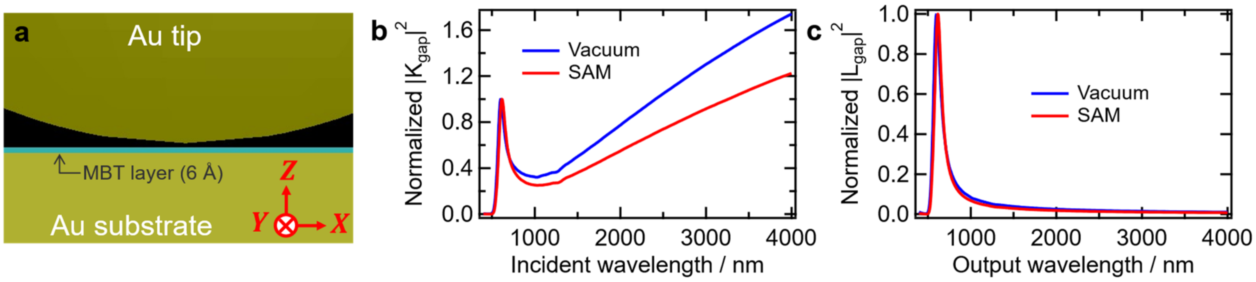

Supplementary Fig. 19 | Theoretical calculation of the near-field properties in the presence of the MBT layer within the gap. a Schematic representation of the gap region including the MBT layer with a thickness of 6 Å and a refractive index of 1.2 (the light blue region). The metal–to–metal separation between the tip apex and the substrate surface was 1 nm. **b, c** The red curves represent the spectra of (**b**) the field enhancement factor ($|K_{\mathrm{gap}}|^2$) and (**c**) the radiation efficiency ($|L_{\mathrm{gap}}|^2$) calculated under the MBT-introduced gap configuration shown in panel **a**. A rounded cone tip with a 30° opening angle, 50 nm radius of curverture, and 15000-nm length was adopted in the calculation. The calculated results obtained without incorporating the MBT layer (the blue curves in **b** and **c**) are also shown as a reference. The data presented in panels **b** and **c** are normalized by the peak values of the gap-mode plasmon resonance at ~620 nm. Note that the blue curves in panels **b** and **c** are identical to the data at l = 15000 nm shown in Supplementary Fig. 16.

Supplementary Note 14. Spatial distributions of electric fields, radiation efficiencies, and charge densities within the gap

In this section, we quantitatively estimate the spatial distribution of the electric field within the nanogap. Supplementary Fig. 20 displays the amplitude mapping of the surface-normal and surface-parallel components of the electric field enhancement factor as a function of spatial position $\mathbf{r}$ ($K_{\text{gap},Z}(\mathbf{r})$ and $K_{\text{gap},\parallel}(\mathbf{r})$, respectively), which were calculated through a three-dimensional FDTD simulation for a tip with a curvature radius of 50 nm. This tip apex radius is comparable to the size of the tip apex used in our experiments (Fig. 1b in the main text). The tip–substrate gap was assumed to be vacuum, and the presence of the SAM layer was not incorporated. Notably, as shown in Supplementary Fig. 20, the enhanced electric field is spatially non-uniform and distributed over the nanoscale region with a radius of ~20 nm. Since the enhancement factor K_{gap} is proportional to E_{gap} ($E_{\text{gap}} = K_{\text{gap}}E_0$), the mappings in Supplementary Fig. 20 directly reflect the spatial distributions of surface-normal and surface-parallel near-field intensities ($E_{\text{gap},Z}(\mathbf{r})$ and $E_{\text{gap},\parallel}(\mathbf{r})$, respectively).

To elucidate the physical origin of the near-field enhancement at the tip–substrate gap, we calculated the charge density distribution in the gap region by applying Gauss's law ($\nabla \cdot E_{\text{gap}} \propto \rho$) to the spatial electric field distribution shown in Supplementary Fig. 20. Supplementary Fig. 21a displays the charge density distribution on the XZ plane (Y = 0 nm) under the 3280-nm excitation, showing that charge neutrality is preserved within the bulk due to screening effects and charges are localized at the metal surfaces. Similar charge distribution is predicted for the near-IR (1033 nm) excitation conditions (Supplementary Fig. 21b). Notably, under these mid- and near-IR excitation conditions, charges of opposite signs are simply distributed across the tip and substrate without forming any multipole-like complex charge distribution. Therefore, the plasmon mode generated under the near-IR irradiation can be regarded as strongly dipolar in nature.

The ~±20-nm length scale of the field variations within the gap (Supplementary Fig. 20) is significantly larger than the size of a single MBT molecule (~6 Å). In such conditions, contributions of quadrupolar and even higher-order molecular polarizations should be minor, and thus the induced nonlinear polarizations in molecules are expected to be predominantly dipolar. Notably, this dipolar dominance has been thoroughly verified in our recent publication by theoretically examining the dipole and quadrupole contributions involved in molecular nonlinear polarizations within an angstrom-scale tip–substrate gap[11]. In this recent study, we also measured vibrationally resonant TE-SFG spectra and demonstrated that $\text{Im}\left(\chi^{(2)}\right)$ signals of surface-adsorbed molecules are governed by dipole-dominated features[11]. Therefore, in the present tip conditions, a dipolar approximation is valid for molecular optical transitions, and the influences of quadrupolar or higher-order polarizations are negligible.

Furthermore, by using the image charge method[55], we also revealed that the electrostatic field across the gap ($E_{\text{DC}}(\mathbf{r})$) also exhibits a similar spatial distribution (Supplementary Figs. 22a and b). This spatial non-uniformity of $E_{\text{DC}}(\mathbf{r})$ (Supplementary Figs. 22a and b) can be attributed to the curved geometry of the tip apex (Supplementary Fig. 22c), unlike a parallel-plate capacitor, where the electrostatic field strength between electrodes is spatially uniform (Supplementary Fig. 22d). Consequently, both $E_{\text{gap}}(\mathbf{r})(= K_{\text{gap}}(\mathbf{r})E_0)$ and $E_{\text{DC}}(\mathbf{r})$ exhibit spatial variations, giving rise to

position-dependent nonlinear polarizations within the nanoscale region beneath the tip apex.

In our TE-SHG/TE-SFG experiments, the detected signal corresponds to the coherent summation of the radiation fields emitted from such position-dependent nonlinear polarizations generated within the gap. As discussed in Supplementary Note 13, the efficiency of this radiation process is described by the factor L_{gap}. Importantly, owing to the curved geometry of the tip apex, the plasmonic enhancement efficiency exhibits position dependence within the gap. As a result, the radiation efficiencies of individual nonlinear polarizations also depend on their positions $\mathbf{r}$: $L_{\mathrm{gap}} \equiv L_{\mathrm{gap}}(\mathbf{r})$. We have examined this source-position dependence of $L_{\mathrm{gap}}(\mathbf{r})$ by numerically calculating the radiation from dipole sources placed at different lateral positions across the surface (Supplementary Fig. 23a). As displayed in Supplementary Fig. 23b, dipole signal radiation primarily occurs in a region with a radius of approximately 10 nm from the tip apex position. Notably, our recent experiments revealed that near-field second-order nonlinear optical signals originate from a nanoscale region with an area of a few tens of nanometers directly beneath the tip apex[11]. Since this result is consistent with theoretically predicted ~10-nm-scale lateral field enhancement areas (Supplementary Figs. 20, 22, and 23), the effective field strength averaged over this ~10-nm region mainly contributes to the generation of near-field second-order nonlinear optical signals.

Based on the spatial distributions and relative intensities of the surface-normal and surface-parallel components, we can directly identify the main nonlinear susceptibility tensor components contributing to the near-field nonlinear generation and the resultant polarization state of emitted light. As shown in Supplementary Fig. 20, the surface-normal (Z-directed) near-field component ($E_{\mathrm{gap},Z}(\mathbf{r})$, Supplementary Figs. 20a and b) is more than one order of magnitude stronger than the surface-parallel field component (Supplementary Figs. 20c and d), indicating that only $E_{\mathrm{gap},Z}(\mathbf{r})$ dominantly contributes to nonlinear optical processes within the gap. In this case, among the 27 tensor components of the second-order nonlinear susceptibility, only $\chi^{(2)}_{XZZ}$, $\chi^{(2)}_{YZZ}$, or $\chi^{(2)}_{ZZZ}$ can in principle contribute to the second-order nonlinear polarization within the tip–substrate gap. When the cylindrical symmetry about the central axis of the tip is satisfied, the contributions of $\chi^{(2)}_{XZZ}$ and $\chi^{(2)}_{YZZ}$ are eliminated, leaving $\chi^{(2)}_{ZZZ}$ as the dominant component[56]. Therefore, the generated second-order nonlinear polarization is mainly oriented along the Z-axis, and the corresponding emitted field is *p*-polarized.

We would like to note that, consistent with this argument, the dominance of $\chi^{(2)}_{ZZZ}$ was also confirmed experimentally in our recent work[11]. By measuring the vibrationally resonant TE-SFG for methyl vibrations in MBT molecules, we revealed that the methyl antisymmetric stretching modes exhibited negative $\mathrm{Im}(\chi^{(2)})$ spectra. Based on the considerations of the explicit values of hyperpolarizability tensors and angular distributions of MBT molecules, $\chi^{(2)}_{ZZZ}$ associated with the methyl antisymmetric modes exhibits negative imaginary part, whereas other hyperpolarizability tensor components, such as $\chi^{(2)}_{XXZ}$ and $\chi^{(2)}_{YYZ}$, are characterized by positive imaginary parts. Therefore, the observation of the negative $\mathrm{Im}(\chi^{(2)})$ indicates that $\chi^{(2)}_{ZZZ}$ component predominantly contributes to the generation of the tip-enhanced second-order nonlinear polarization. Therefore, not only theoretical analysis but also our experimental results[11] consistently corroborates the predominance of

$\chi^{(2)}_{ZZZ}$, indicating that near-field nonlinear signal radiation is dominantly *p*-polarized.

Similar to the second-order case, the third-order polarization is also oriented along the *Z*-axis. In addition to the oscillating near-field induced by the incident excitation light (Supplementary Fig. 20), the electrostatic field generated through the voltage application across the tip–substrate gap is also dominated by its *Z*-component (Supplementary Fig. 22). Therefore, by analogy with the second-order susceptibility, only $\chi^{(3)}_{ZZZZ}$ component is active among all third-order susceptibility tensor components. This ensures that the third-order nonlinear polarization in EFISH is also aligned along the *Z*-axis and that the corresponding emitted field is *p*-polarized.

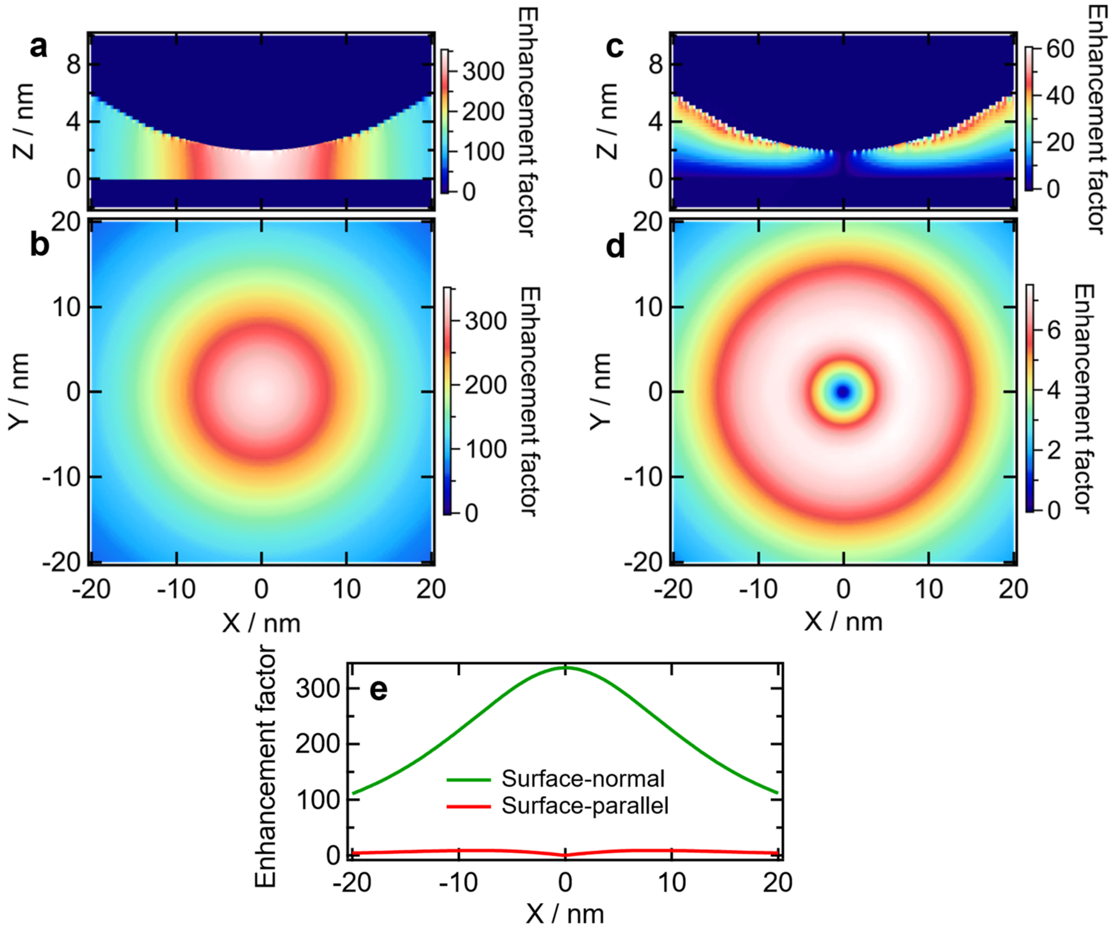

Supplementary Fig. 20 | Spatial distributions of the field enhancement factor K_{gap}. The theoretical predictions of (**a, b**) surface-normal and (**c, d**) surface-parallel electric field components calculated through the FDTD method are displayed. The model employs a rounded gold cone with a 30° opening angle, 50-nm apex radius, and 15000-nm length. The tip–substrate distance was set to be 2 nm. The calculation was performed in three-dimensional simulation space. Panels (**a**) and (**c**) show the field in the *XZ* plane (*Y* = 0 nm), while panels (**b**) and (**d**) show the field in the *XY* plane (*Z* = 0.5 nm). The wavelength of incident light was 3280 nm. The coordinates (*X*, *Y*) = (0 nm, 0 nm) represent the position of minimum tip–substrate distance. **e** One dimensional spatial profiles of K_{gap} along the *X*-axis (*Y* = 0 nm and *Z* = 0.6 nm). Green and red curves represent the surface-normal and surface-parallel field components, respectively.

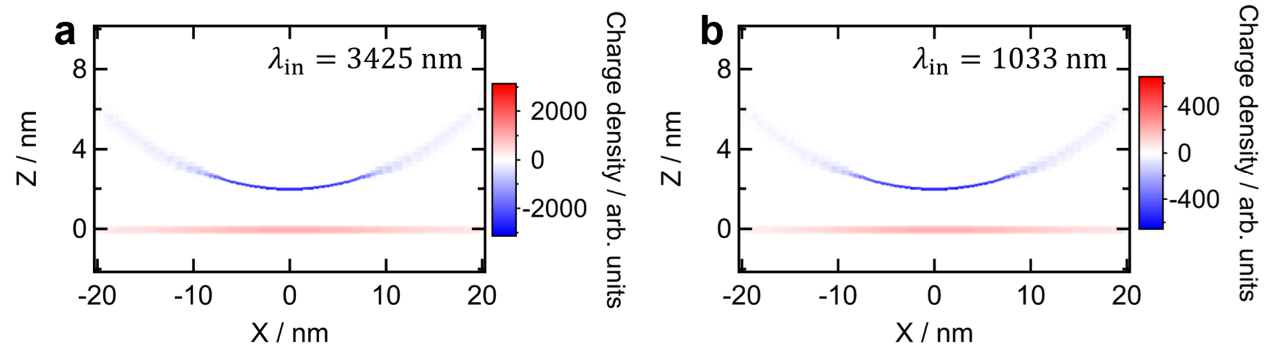

Supplementary Fig. 21 | Charge distribution across the tip and substrate on the *XZ* plane (*Y* = 0 nm) under the IR excitation. These charge densities were obtained by applying Gauss's law ($\nabla \cdot E_{gap} \propto \rho$) to the spatial electric field distribution. Panels **a** and **b** represent the Fourier component corresponding to 3280-nm and 1033-nm excitation, respectively. The bulk region is electrically neutral due to the screening effects, and charges with opposite signs are accumulated at the metal surfaces.

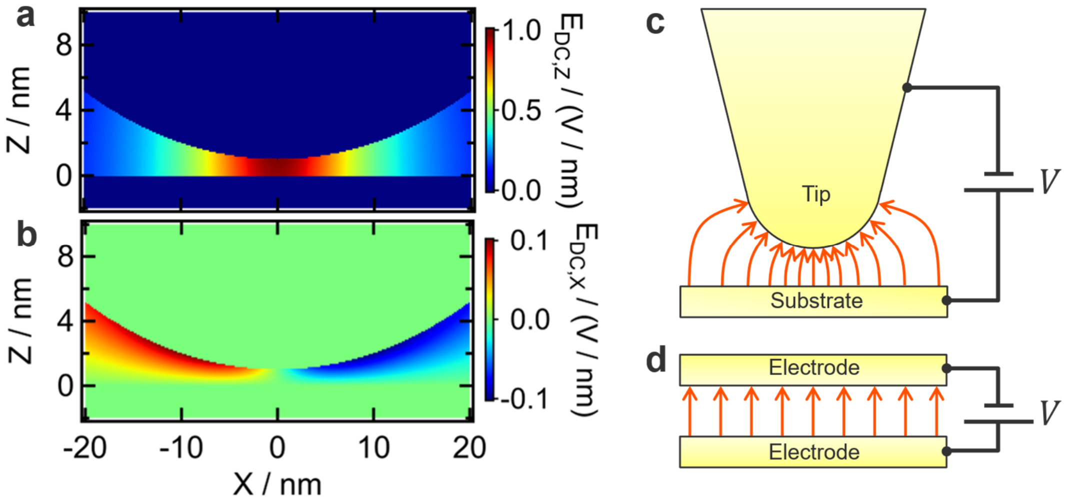

Supplementary Fig. 22 | Spatial distributions of the electrostatic field E_{DC} across the tip-substrate nanogap. a, b The surface-normal (**a**) and surface-parallel (**b**) electrostatic field components calculated through the image charge method[55] are plotted in the *XZ* plane (*Y* = 0 nm). In the calculation, the tip was approximated by a nanosphere with a 50-nm apex radius, the tip–substrate distance was set to be 1 nm, and the applied bias was assumed to be 1 V. The coordinates (*X, Y*) = (0 nm, 0 nm) represent the position of minimum tip–substrate distance. **c, d** Schematic illustrations of electrostatic field distributions within a tip–substrate nanogap and (**c**) a parallel-plate capacitor (**d**) under an applied bias V. The orange arrows indicate the electric flux lines within the gaps, illustrating non-uniform field distribution in the tip–substrate nanogap arising from the curved tip geometry (**c**) and uniform field distribution in the parallel-plate geometry (**d**).

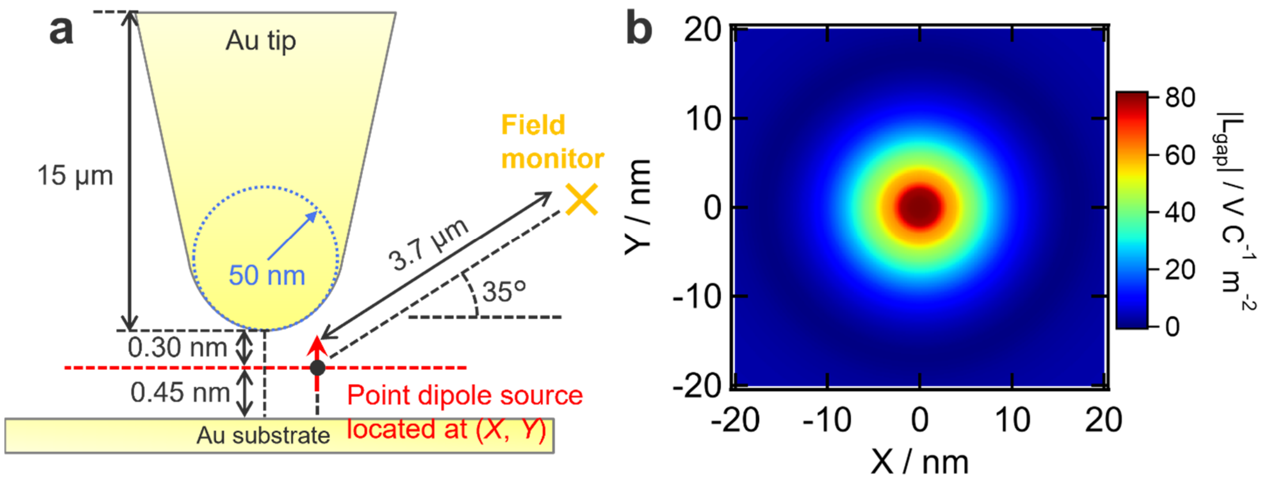

Supplementary Fig. 23 | Spatial properties of the radiation efficiency $L_{gap}(\mathbf{r})$. a Schematic illustration of the calculation. Electric fields emitted from the gap region were monitored for different point-dipole source positions along the red broken horizontal line, yielding a one-dimensional profile of source position dependence of L_{gap}. To reduce the computational cost, the calculation region was restricted to a two-dimensional plane perpendicular to the substrate surface and containing the point dipole source and the field monitor. **b** Source position dependence of $L_{gap}(\mathbf{r})$ at an emission wavelength of 750 nm, corresponding to the central wavelength of TE-SHG signal (Fig. 2 in the main text). This two-dimensional spatial mapping was obtained by rotating the one-dimensional source-position-dependent profile of L_{gap} about the Z-axis.

Supplementary Note 15. Influences of spatial distributions of electric fields on near-field nonlinear optical effects

In Supplementary Note 13, we overviewed the spectral properties of both the field enhancement factor within the tip–substrate gap (K_{gap}) and the radiation efficiency from a single emitter placed within the gap region (L_{gap}). Although the discussion in Supplementary Note 13 provides physically important interpretation regarding the fundamental mechanisms of TE-SHG and TE-SFG processes, it still disregards the presence of the inherent spatial non-uniformity in K_{gap}, L_{gap}, and E_{DC} demonstrated in Supplementary Note 14. In the actual tip–substrate gap, spatially varying K_{gap} and E_{DC} (Supplementary Figs. 20 and 22, respectively) produce position-dependent nonlinear polarizations within the nanoscale region, and radiation from each polarization occurs with different efficiencies depending on its absolute position (Supplementary Fig. 23). The experimentally observed near-field nonlinear optical signals correspond to the coherent sum of these individual contributions. Here, we demonstrate that even in the presence of such spatial distributions, the overall TE-SHG intensity can be reduced to the simple form given in Eq. 1 in the main text by considering the spatially averaged signal intensity within the near-field enhancement region.

The overall TE-SHG intensity (I_{TESHG}), including the contributions from both the bias-independent second-order field and E_{DC}-induced third-order field, is given by the total intensity of SHG fields emitted from N molecules within the gap:

$$I_{\mathrm{TESHG}} \propto \left|E_{\mathrm{TESHG}}^{(2)} + E_{\mathrm{TESHG}}^{(3)}\right|^2$$
$$= N^2 \left|\chi^{(2)}\left\langle L_{\mathrm{gap}}(\mathbf{r}_{\mathrm{n}}) K_{\mathrm{gap},z}(\mathbf{r}_{\mathrm{n}})^2\right\rangle + \chi^{(3)}\left\langle \frac{L_{\mathrm{gap}}(\mathbf{r}_{\mathrm{n}}) K_{\mathrm{gap},z}(\mathbf{r}_{\mathrm{n}})^2}{d(\mathbf{r}_{\mathrm{n}})}\right\rangle V\right|^2 I_0^2, \quad (5)$$

where $E_{\mathrm{TESHG}}^{(2)}$ and $E_{\mathrm{TESHG}}^{(3)}$ represent the electric fields of the DC-field-independent second-order TE-SHG signal and DC-field-induced third-order TE-SHG signal; $\langle\cdots\rangle$ denotes spatial averaging over the molecules present within the gap region; $\chi^{(2)}$ and $\chi^{(3)}$ are second- and third-order nonlinear susceptibilities, respectively; $\mathbf{r}_{\mathrm{n}}$ is the position of n-th molecule; $d(\mathbf{r}_{\mathrm{n}})$ represents the position-dependent tip–substrate distance determined by the curvature radius of the tip apex; V denotes the applied STM bias voltage; and I_0 is incident field intensity. To simplify this formulation, we introduce an effective tip-substrate distance d_{eff}, which satisfies the relation

$$\left\langle \frac{L_{\mathrm{gap}}(\mathbf{r}_{\mathrm{n}}) K_{\mathrm{gap},z}(\mathbf{r}_{\mathrm{n}})^2}{d(\mathbf{r}_{\mathrm{n}})}\right\rangle = \frac{\left\langle L_{\mathrm{gap}}(\mathbf{r}_{\mathrm{n}}) K_{\mathrm{gap},z}(\mathbf{r}_{\mathrm{n}})^2\right\rangle}{d_{\mathrm{eff}}}. \quad (6)$$

Substituting this expression into Supplementary Equation (5) yields

$$I_{\mathrm{TESHG}} \propto N^2 \left|\chi^{(2)} + \chi^{(3)}\left(\frac{V}{d_{\mathrm{eff}}}\right)\right|^2 \left|\left\langle L_{\mathrm{gap}}(\mathbf{r}_{\mathrm{n}}) K_{\mathrm{gap},z}(\mathbf{r}_{\mathrm{n}})^2\right\rangle\right|^2 I_0^2. \quad (7)$$

Then, we define V/d_{eff} term as $E_{\mathrm{DC,eff}}$, which corresponds to a spatially averaged effective intragap electrostatic field incorporating both the non-uniform field distribution in the gap and the curved geometry of the tip apex. Furthermore, we introduce an effective radiation efficiency $L_{\mathrm{gap,eff}}$ defined as

$$\left\langle L_{\mathrm{gap}}(\mathbf{r}_{\mathrm{n}}) K_{\mathrm{gap},z}(\mathbf{r}_{\mathrm{n}})^2\right\rangle = L_{\mathrm{gap,eff}}\left\langle K_{\mathrm{gap},z}(\mathbf{r}_{\mathrm{n}})^2\right\rangle, \quad (8)$$

where $L_{\text{gap,eff}}$ corresponds to the weighted average of $L_{\text{gap}}(\mathbf{r}_\text{n})$ by $K_{\text{gap},Z}(\mathbf{r}_\text{n})^2$. Using these two effective values ($E_{\text{DC,eff}}$ and $L_{\text{gap,eff}}$) yields

$$I_{\text{TESHG}} \propto N^2 \left|L_{\text{gap,eff}}\right|^2 \left|\chi^{(2)} + \chi^{(3)} E_{\text{DC,eff}}\right|^2 \left|\langle K_{\text{gap},Z}(\mathbf{r}_\text{n})^2\rangle\right|^2 I_0{}^2. \quad (9)$$

This equation shows that the spatially averaged near-field intensity within the gap should be given by mean-square intensity:

$$\langle I_{\text{gap}}\rangle = \left|\langle K_{\text{gap},Z}(\mathbf{r}_\text{n})^2\rangle\right| I_0. \quad (10)$$

Using this average near-field intensity and omitting $\left|L_{\text{gap,eff}}\right|^2$ constant from Supplementary Equation (9) for simplicity, we arrive at the simplified expression for EFISH similar to that given in Eq. 1 in the main text:

$$I_{\text{TESHG}} \propto \left|\chi^{(2)} + \chi^{(3)} E_{\text{DC,eff}}\right|^2 \langle I_{\text{gap}}\rangle^2. \quad (11)$$

Notably, while this equation incorporates the spatial distributions of both the optical near field and the electrostatic field, it is still described by a formally equivalent expression for the EFISH effect under homogeneous electrostatic field. Moreover, even within this refined formalism, $E_{\text{DC,eff}}$ is still proportional to V ($E_{\text{DC,eff}} = V/d_{\text{eff}}$), and thus I_{TESHG} in Supplementary Equation (11) quadratically depends on V. This validates the fitting analysis using a quadratic function presented in Figs. 3 and 4e in the main text.

Based on the scanning electron micrograph of the tip apex structure (Fig. 1b in the main text) and the field distribution analyses shown in Supplementary Note 14, we can estimate the explicit value of d_{eff}. The bias-dependent TE-SHG/TE-SFG measurements (Figs. 3, 4d, and 4e in the main text) were performed by using a tip with ~50-nm apex curvature (Fig. 1b in the main text), and the tip–substrate distance d employed in those experiments was ~7 Å. In such conditions, the effective tip–substrate distance d_{eff}, calculated by substituting the spatial distributions of $K_{\text{gap},Z}(\mathbf{r}_\text{n})$ (Supplementary Figs. 20a and b) and the source position dependence of $L_{\text{gap}}(\mathbf{r}_\text{n})$ (Supplementary Fig. 23b) into Supplementary Equation (6), is approximately 9.7 Å, indicating that d_{eff} is larger than d by ~2.7 Å. This d_{eff} value was used to obtain the estimates of the relative values of $\chi^{(2)}$ and $\chi^{(3)}$ presented in the main text.

As shown in Supplementary Fig. 11d, the excitation light irradiation at the tip apex induces ~0.5-Å fluctuations in the tip–substrate distance d. These fluctuations lead to corresponding variations in both the effective distance d_{eff} and the effective electrostatic field $E_{\text{DC,eff}}$, resulting in temporal fluctuations in the overall TE-SHG intensity. To quantitatively examine the influences of such fluctuations, we additionally calculated d_{eff} values for d = 6.5 Å and 7.5 Å conditions and obtained d_{eff} = 9.2 Å and 10.2 Å, respectively. Therefore, when the tip–substrate distance d fluctuates by ±0.5 Å around 7 Å (Supplementary Fig. 11d), the effective tip–substrate distance d_{eff} also undergoes ±0.5-Å fluctuations around its average value of 9.7 Å. The influences of such variations in d_{eff} were examined by numerically calculating the relative modulation of the TE-SHG intensity ($\Delta I_{\text{TESHG}}(V)/I_{\text{TESHG}}(V=0)$, Eq. 5 in the main text) for three effective gap distances of d_{eff} = 9.2, 9.7, and 10.2 Å (Supplementary Fig. 24). Notably, within such distance range around 9.7 Å, the distance-dependent variations in modulation depth are sufficiently small compared with the overall ~2000% modulation (Supplementary Fig. 24). This is because the amplitude of the distance

variation (±0.5 Å) is sufficiently smaller than the average value of d_{eff} (9.7 Å), leading to only minor variations in the coefficient of V^2 term in Eq. 5 in the main text ($\left|\chi^{(3)}\right|^2/\left|\chi^{(2)}\right|^2 d_{\text{eff}}^2$). Therefore, sub-angstrom fluctuations in the gap distance have an only minor influence on the measured electrophotonic modulation. Additionally, as described in the main text, the timescale of the distance fluctuations during the measurements is on the order of microsecond scale, substantially shorter than minute-scale signal integration time in our measurements. Consequently, the influence of the fluctuations in the effective electrostatic field ($E_{\text{DC,eff}}$) would be time-averaged and would not manifest in the measured modulation curves. Based on these considerations, we can clearly rule out the possibility that ±0.5-Å variations in d (Supplementary Fig. 11d) affect the observed giant field-induced modulation behaviors.

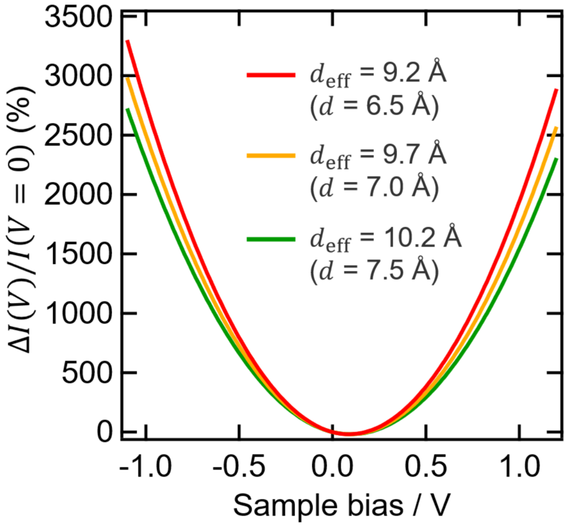

Supplementary Fig. 24 | Numerical analysis of gap distance sensitivity. Calculated bias-dependent relative modulations of TE-SHG intensities (Eq. 5 in the main text) for effective gap distances d_{eff} of 9.2 Å (red), 9.7 Å (orange), and 10.2 Å (green), corresponding to d = 6.5, 7.0, 7.5 Å, respectively.

Supplementary References

1. Horcas, I. *et al.* WSXM : A software for scanning probe microscopy and a tool for nanotechnology. *Rev. Sci. Instrum.* **78**, 013705 (2007).
2. Hong, M., Yokota, Y., Hayazawa, N., Kazuma, E. & Kim, Y. Homogeneous Dispersion of Aromatic Thiolates in the Binary Self-Assembled Monolayer on Au(111) via Displacement Revealed by Tip-Enhanced Raman Spectroscopy. *J. Phys. Chem. C* **124**, 13141–13149 (2020).
3. Yang, G. & Liu, G. New Insights for Self-Assembled Monolayers of Organothiols on Au(111) Revealed by Scanning Tunneling Microscopy. *J. Phys. Chem. B* **107**, 8746–8759 (2003).
4. Yokota, Y. *et al.* Systematic Assessment of Benzenethiol Self-Assembled Monolayers on Au(111) as a Standard Sample for Electrochemical Tip-Enhanced Raman Spectroscopy. *J. Phys. Chem. C* **123**, 2953–2963 (2019).
5. Okabayashi, N., Konda, Y. & Komeda, T. Inelastic Electron Tunneling Spectroscopy of an Alkanethiol Self-Assembled Monolayer Using Scanning Tunneling Microscopy. *Phys. Rev. Lett.* **100**, 217801 (2008).
6. Bumm, L. A., Arnold, J. J., Dunbar, T. D., Allara, D. L. & Weiss, P. S. Electron Transfer through Organic Molecules. *J. Phys. Chem. B* **103**, 8122–8127 (1999).
7. Fujii, S., Ziatdinov, M., Higashibayashi, S., Sakurai, H. & Kiguchi, M. Bowl Inversion and Electronic Switching of Buckybowls on Gold. *J. Am. Chem. Soc.* **138**, 12142–12149 (2016).
8. Yang, B. *et al.* Chemical Enhancement and Quenching in Single-Molecule Tip-Enhanced Raman Spectroscopy. *Angew. Chem. Int. Ed.* **62**, e202218799 (2023).
9. Chen, C. J. *Introduction to Scanning Tunneling Microscopy*. (Oxford University Press, 1994).
10. Seo, K. & Borguet, E. Potential-Induced Structural Change in a Self-Assembled Monolayer of 4-Methylbenzenethiol on Au(111). *J. Phys. Chem. C* **111**, 6335–6342 (2007).
11. Takahashi, S. *et al.* Tip-Enhanced Sum-Frequency Vibrational Nanoscopy beyond the Diffraction Limit. *J. Phys. Chem. C* **130**, 373–385 (2026).
12. Domke, K. F., Zhang, D. & Pettinger, B. Toward Raman Fingerprints of Single Dye Molecules at Atomically Smooth Au(111). *J. Am. Chem. Soc.* **128**, 14721–14727 (2006).
13. Haiss, W., Lackey, D., Sass, J. K. & Besocke, K. H. Atomic resolution scanning tunneling microscopy images of Au(111) surfaces in air and polar organic solvents. *J. Chem. Phys.* **95**, 2193–2196 (1991).
14. Barth, J. V., Brune, H., Ertl, G. & Behm, R. J. Scanning tunneling microscopy observations on the reconstructed Au(111) surface: Atomic structure, long-range superstructure, rotational domains, and surface defects. *Phys. Rev. B* **42**, 9307–9318 (1990).
15. Fu, Y., Zhang, P., Verboncoeur, J. P. & Wang, X. Electrical breakdown from macro to micro/nano scales: a tutorial and a review of the state of the art. *Plasma Res. Express* **2**, 013001 (2020).
16. Liu, S. *et al.* Inelastic Light Scattering in the Vicinity of a Single-Atom Quantum Point Contact in a Plasmonic Picocavity. *ACS Nano* **17**, 10172–10180 (2023).
17. Zuloaga, J., Prodan, E. & Nordlander, P. Quantum Description of the Plasmon Resonances of a Nanoparticle Dimer. *Nano Lett.* **9**, 887–891 (2009).

18. Savage, K. J. *et al.* Revealing the quantum regime in tunnelling plasmonics. *Nature* **491**, 574–577 (2012).
19. Esteban, R., Borisov, A. G., Nordlander, P. & Aizpurua, J. Bridging quantum and classical plasmonics with a quantum-corrected model. *Nat. Commun.* **3**, 825 (2012).
20. Zhu, W. & Crozier, K. B. Quantum mechanical limit to plasmonic enhancement as observed by surface-enhanced Raman scattering. *Nat. Commun.* **5**, 5228 (2014).
21. Marinica, D. C., Kazansky, A. K., Nordlander, P., Aizpurua, J. & Borisov, A. G. Quantum Plasmonics: Nonlinear Effects in the Field Enhancement of a Plasmonic Nanoparticle Dimer. *Nano Lett.* **12**, 1333–1339 (2012).
22. Zhu, W. *et al.* Quantum mechanical effects in plasmonic structures with subnanometre gaps. *Nat. Commun.* **7**, 11495 (2016).
23. Takahashi, S., Sakurai, A., Mochizuki, T. & Sugimoto, T. Broadband Tip-Enhanced Nonlinear Optical Response in a Plasmonic Nanocavity. *J. Phys. Chem. Lett.* **14**, 6919–6926 (2023).
24. De Luca, F. & Ciracì, C. Impact of Surface Charge Depletion on the Free Electron Nonlinear Response of Heavily Doped Semiconductors. *Phys. Rev. Lett.* **129**, 123902 (2022).
25. Downes, A., Salter, D. & Elfick, A. Finite Element Simulations of Tip-Enhanced Raman and Fluorescence Spectroscopy. *J. Phys. Chem. B* **110**, 6692–6698 (2006).
26. Chen, X. & Wang, X. Near-field thermal transport in a nanotip under laser irradiation. *Nanotechnology* **22**, 075204 (2011).
27. Esteban, R. *et al.* A classical treatment of optical tunneling in plasmonic gaps: extending the quantum corrected model to practical situations. *Faraday Discuss.* **178**, 151–183 (2015).
28. Martín-Jiménez, A. *et al.* Unveiling the radiative local density of optical states of a plasmonic nanocavity by STM. *Nat. Commun.* **11**, 1021 (2020).
29. Sakurai, A., Takahashi, S., Mochizuki, T. & Sugimoto, T. Tip-Enhanced Sum Frequency Generation for Molecular Vibrational Nanospectroscopy. *Nano Lett.* **25**, 6390–6398 (2025).
30. Kane Yee. Numerical solution of initial boundary value problems involving maxwell's equations in isotropic media. *IEEE Trans. Antennas Propagat.* **14**, 302–307 (1966).
31. Anees, A. & Angermann, L. Time Domain Finite Element Method for Maxwell's Equations. *IEEE Access* **7**, 63852–63867 (2019).
32. Teixeira, F. L. *et al.* Finite-difference time-domain methods. *Nat. Rev. Methods Primers* **3**, 75 (2023).
33. Olmon, R. L. *et al.* Optical dielectric function of gold. *Phys. Rev. B* **86**, 235147 (2012).
34. Jaculbia, R. B. *et al.* Single-molecule resonance Raman effect in a plasmonic nanocavity. *Nat. Nanotechnol.* **15**, 105–110 (2020).
35. Heilman, A. L., Hermann, R. J. & Gordon, M. J. Direct detection of gap mode plasmon resonances using attenuated total reflection-based tip-enhanced near-field optical microscopy. *J. Opt.* **22**, 095001 (2020).
36. Porto, J. A., Johansson, P., Apell, S. P. & López-Ríos, T. Resonance shift effects in apertureless scanning near-field optical microscopy. *Phys. Rev. B* **67**, 085409 (2003).

37. Krug, J. T., Sánchez, E. J. & Xie, X. S. Design of near-field optical probes with optimal field enhancement by finite difference time domain electromagnetic simulation. *J. Chem. Phys.* **116**, 10895–10901 (2002).
38. Madrazo, A., Nieto-Vesperinas, M. & García, N. Exact calculation of Maxwell equations for a tip-metallic interface configuration: Application to atomic resolution by photon emission. *Phys. Rev. B* **53**, 3654–3657 (1996).
39. Esteban, R., Vogelgesang, R. & Kern, K. Tip-substrate interaction in optical near-field microscopy. *Phys. Rev. B* **75**, 195410 (2007).
40. Li, G. *et al.* Plasmonic enhancement and directional emission for side-illumination tip-enhanced spectroscopy. *Opt. Commun.* **442**, 50–55 (2019).
41. Wei, Y., Pei, H., Sun, D., Duan, S. & Tian, G. Numerical investigations on the electromagnetic enhancement effect to tip-enhanced Raman scattering and fluorescence processes. *J. Phys.: Condens. Matter* **31**, 235301 (2019).
42. Futamata, M., Ishikura, M., Iida, C. & Handa, S. The critical importance of gap modes in surface enhanced Raman scattering. *Faraday Discuss.* **178**, 203–220 (2015).
43. Huth, F. *et al.* Resonant Antenna Probes for Tip-Enhanced Infrared Near-Field Microscopy. *Nano Lett.* **13**, 1065–1072 (2013).
44. Mastel, S. *et al.* Terahertz Nanofocusing with Cantilevered Terahertz-Resonant Antenna Tips. *Nano Lett.* **17**, 6526–6533 (2017).
45. Hermann, R. J. & Gordon, M. J. Quantitative comparison of plasmon resonances and field enhancements of near-field optical antennae using FDTD simulations. *Opt. Express* **26**, 27668–27682 (2018).
46. Zhang, W., Cui, X. & Martin, O. J. F. Local field enhancement of an infinite conical metal tip illuminated by a focused beam. *J. Raman Spectrosc.* **40**, 1338–1342 (2009).
47. Sanders, A. *et al.* Understanding the plasmonics of nanostructured atomic force microscopy tips. *Appl. Phys. Lett.* **109**, 153110 (2016).
48. Berweger, S., Atkin, J. M., Olmon, R. L. & Raschke, M. B. Light on the Tip of a Needle: Plasmonic Nanofocusing for Spectroscopy on the Nanoscale. *J. Phys. Chem. Lett.* **3**, 945–952 (2012).
49. Giugni, A. *et al.* Adiabatic nanofocusing: spectroscopy, transport and imaging investigation of the nano world. *J. Opt.* **16**, 114003 (2014).
50. Schmucker, S. W. *et al.* Field-directed sputter sharpening for tailored probe materials and atomic-scale lithography. *Nat. Commun.* **3**, 935 (2012).
51. Böckmann, H. *et al.* Near-Field Manipulation in a Scanning Tunneling Microscope Junction with Plasmonic Fabry-Pérot Tips. *Nano Lett.* **19**, 3597–3602 (2019).
52. Zhuang, X., Miranda, P. B., Kim, D. & Shen, Y. R. Mapping molecular orientation and conformation at interfaces by surface nonlinear optics. *Phys. Rev. B* **59**, 12632–12640 (1999).
53. Dalstein, L., Revel, A., Humbert, C. & Busson, B. Nonlinear optical response of a gold surface in the visible range: A study by two-color sum-frequency generation spectroscopy. I. Experimental

determination. *J. Chem. Phys.* **148**, 134701 (2018).

54. Fellows, A. P. *et al.* Obtaining extended insight into molecular systems by probing multiple pathways in second-order nonlinear spectroscopy. *J. Chem. Phys.* **159**, 164201 (2023).
55. Dall'Agnol, F. F. & Mammana, V. P. Solution for the electric potential distribution produced by sphere-plane electrodes using the method of images. *Rev. Bras. Ensino Fís.* **31**, 3503.1-3503.9 (2009).
56. Wang, H.-F., Gan, W., Lu, R., Rao, Y. & Wu, B.-H. Quantitative spectral and orientational analysis in surface sum frequency generation vibrational spectroscopy (SFG-VS). *Int. Rev. Phys. Chem.* **24**, 191–256 (2005).
